# Comparative genomics of the *Bifidobacterium breve* taxon

**DOI:** 10.1186/1471-2164-15-170

**Published:** 2014-03-01

**Authors:** Francesca Bottacini, Mary O’Connell Motherway, Justin Kuczynski, Kerry Joan O’Connell, Fausta Serafini, Sabrina Duranti, Christian Milani, Francesca Turroni, Gabriele Andrea Lugli, Aldert Zomer, Daria Zhurina, Christian Riedel, Marco Ventura, Douwe van Sinderen

**Affiliations:** Alimentary Pharmabiotic Centre and Department of Microbiology, Bioscience Institute, National University of Ireland, Western Road, Cork, Ireland; Laboratory of Probiogenomics, Department of Life Sciences, University of Parma, Parma, Italy; Second Genome, Bioinformatics Department, San Bruno, CA USA; Centre for Molecular and Biomolecular Informatics, Nijmegen Centre for Molecular Life Sciences, Radboud University Medical Centre, Nijmegen, The Netherlands; Institute of Microbiology and Biotechnology, University of Ulm, Ulm, Germany

**Keywords:** *Bifidobacterium breve*, Evolutionary genomics, Core genome, Dispensable genome, Pan-genome

## Abstract

**Background:**

Bifidobacteria are commonly found as part of the microbiota of the gastrointestinal tract (GIT) of a broad range of hosts, where their presence is positively correlated with the host’s health status. In this study, we assessed the genomes of thirteen representatives of *Bifidobacterium breve*, which is not only a frequently encountered component of the (adult and infant) human gut microbiota, but can also be isolated from human milk and vagina.

**Results:**

*In silico* analysis of genome sequences from thirteen *B. breve* strains isolated from different environments (infant and adult faeces, human milk, human vagina) shows that the genetic variability of this species principally consists of hypothetical genes and mobile elements, but, interestingly, also genes correlated with the adaptation to host environment and gut colonization. These latter genes specify the biosynthetic machinery for sortase-dependent pili and exopolysaccharide production, as well as genes that provide protection against invasion of foreign DNA (i.e. CRISPR loci and restriction/modification systems), and genes that encode enzymes responsible for carbohydrate fermentation. Gene-trait matching analysis showed clear correlations between known metabolic capabilities and characterized genes, and it also allowed the identification of a gene cluster involved in the utilization of the alcohol-sugar sorbitol.

**Conclusions:**

Genome analysis of thirteen representatives of the *B. breve* species revealed that the deduced pan-genome exhibits an essentially close trend. For this reason our analyses suggest that this number of *B. breve* representatives is sufficient to fully describe the pan-genome of this species. Comparative genomics also facilitated the genetic explanation for differential carbon source utilization phenotypes previously observed in different strains of *B. breve*.

**Electronic supplementary material:**

The online version of this article (doi:10.1186/1471-2164-15-170) contains supplementary material, which is available to authorized users.

## Background

Bifidobacteria are a common component of the microbiota of the gastrointestinal tract (GIT) of a broad range of hosts, and their presence is associated with a positive health status of the gut [1]. However, little is known about the precise molecular mechanisms that explain these probiotic effects [1–3]. For this reason a considerable number of ongoing scientific efforts aim to precisely explain how these benefits are being provided, and in many cases such efforts involve comparative and functional genome analyses.

Sequenced bifidobacterial genomes range in size from 1.94 to 2.8 Mbp (*Bifidobacterium animalis* subsp. *lactis* DSM 10140 and *Bifidobacterium longum* subsp. *infantis* ATCC 15697, respectively), and their genomic organization is in line with that of a typical bacterial chromosome [4].

*B. longum* subsp. *infantis*, *Bifidobacterium bifidum* and *Bifidobacterium breve* are typical inhabitants of the infant intestine, which is presumed sterile at birth but becomes rapidly colonized by bacteria immediately following (vaginal) delivery [5, 6]. Functional analyses conducted on bifidobacterial genomes have also revealed how they adapt to a certain niche. For example, the presence of enzymes dedicated to the metabolism of human milk oligosaccharides (HMOs) in *B. longum* subsp. *infantis* showed how this species is specialized in colonizing the infant gut [7].

*In vivo* gene expression analyses conducted on *B. breve* UCC2003 and *B. bifidum* PRL2010 have revealed genes that encode functions required for gut colonization and persistence [8, 9]. Furthermore, Comparative Genome Hybridization (CGH) analyses on various *B. breve* isolates has highlighted the existence of a high level of sequence homology among members of this species, and it also identified genetic functions that appear to be more variable within this bifidobacterial taxon [8]. Such variable functions are associated with bifidobacterial adaptation to the host environment and defence against invasion of foreign DNA. They include, among others, CRISPR (Clustered Regularly Interspaced Short Palindromic Repeats) sequences, (type II) Restriction/Modification (R/M) systems and genes involved in the production of particular extracellular structures, such as capsule exopolysaccharides (EPS) and sortase-dependent pili [8]. However, CGH analysis is not sufficient to describe the genetic diversity of a species, as it can only detect genes present in the reference genome, but cannot identify genes that are present in tested genomes yet absent in the reference genome. For this reason we decided to investigate the genome variability within the *B. breve* taxon by performing whole genome sequencing and comparative analysis of thirteen *B. breve* strains. The generated genome data sets were used to perform a pan-genomic analysis which allowed the definition of the total number of different genes encoded by the entire *B. breve* group (the pan-genome), as well as the total number of common genes present in all isolates (the core-genome) [10, 11]. Corresponding pan- and core-genome information, as obtained by an increasing number of genome sequences, can be used to determine if sequenced representatives of a certain species have provided all expected gene diversity present in that taxon (closed trend), or if additional sequencing is still necessary before essentially all genes of the species have been identified (open trend) [10–12]. As this approach takes the overall collection of genetic functions assigned to a certain species (pan-genome) in consideration, rather than conducting individual analyses for each strain, it is believed to represent an accurate and advanced method to explore genomic diversity of a particular bacterial taxon.

## Results and discussion

### General genome features

In order to assess the chromosomal features of representative members of the *B. breve* species, we analyzed the genome sequences of thirteen different *B. breve* strains (Table 1), eight of which were sequenced in the framework of this study (*B. breve* 689b, *B. breve* 12L, *B. breve* 2L, *B. breve* 31L, *B. breve* NCFB 2258, *B. breve* S27, *B. breve* JCM 7017, *B. breve* JCM 7019), and which had previously been isolated from different human environments, such as infant feaces (*B. breve* 689b, *B. breve* NCFB 2258, *B. breve* S27, *B. breve* JCM 7017), human milk (*B. breve* 12L, *B. breve* 2L, *B. breve* 31L) or adult faeces (*B. breve* JCM 7019).

**Table 1:** **List of** ***Bifidobacterium breve*** **representatives**

***Strain name***	***Ecological origin***	***Affilation***	***Accession number***	***Sequencing status***
***B. breve*** **UCC2003**	Infant faeces	University College Cork, Ireland	NC_020517	COMPLETE
***B. breve*** **S27**	Infant feces (breast fed)	University of Ulm, Germany	CP006716	COMPLETE
***B. breve*** **689b**	Infant faeces	University of Parma, Italy	CP006715	COMPLETE
***B. breve*** **NCFB 2258**	Infant faeces	National Collection of Food Bacteria, UK	CP006714	COMPLETE
***B. breve*** **JCM 7017**	Infant faeces	Japan Collection of Microorgnisms, Japan	CP006712	COMPLETE
***B. breve*** **DSM 20213**	Infant intestine	Deutsche Sammlung von Mikroorganismen, Germany	ACCG00000000	DRAFT (103 contigs)
***B. breve*** **12L**	Human milk	University of Parma, Italy	CP006711	COMPLETE
***B. breve*** **2L**	Human milk	GenProbio Ltd., Parma, Italy	AWUG00000000	DRAFT (6 contigs)
***B. breve*** **31L**	Human milk	GenProbio Ltd., Parma, Italy	AWUF00000000	DRAFT (4 contigs)
***B. breve*** **CECT 7263**	Human milk	Universidad de Madrid, Spain	AFVV00000000	DRAFT (34 contigs)
***B. breve*** **JCM 7019**	Adult faeces	Japan Collection of Microorgnisms, Japan	CP006713	COMPLETE
***B. breve*** **DPC 6330**	Elderly individual faecal sample	Food Research Centre, Moorepark, Cork, Ireland	AFXX00000000	DRAFT (47 contigs)
***B. breve*** **ACS-071-V-Sch8b**	Human vagina	Craig Venter Institute, USA	NC_017218	COMPLETE

Our sequencing efforts resulted in fully sequenced genomes for six strains (*B. breve* 689b, *B. breve* 12L, *B. breve* NCFB 2258, *B. breve* S27, *B. breve* JCM 7017 and *B. breve* JCM 7019), while the assembly of the two remaining genome sequences resulted in multiple contigs (Table 1). Furthermore, five complete and draft *B. breve* genomes (*B. breve* UCC2003, *B. breve* ACS-071-V-Sch8b, *B. breve* CECT 7263, *B. breve* DPC 6330, *B. breve* DSM 20213) were retrieved from the NCBI public database. Genome alignment conducted on the eight complete genomes, using *B. breve* UCC2003 as reference sequence [8], established an average sequence length of 2,323,100 bp, where *B. breve* JCM 7017 represents the strain with the smallest chromosome (with a size of 2,288,919 bp), while *B. breve* UCC2003 possessed the largest chromosome (with a size of 2,422,684). All *B. breve* genomes here analyzed displayed an average G+C content of 58%, which is consistent with the range of G+C mol% content of genomes of the *Bifidobacterium* genus [12].

In order to facilitate a coherent comparative analysis, we performed a consistent open reading frame (ORF) prediction for all available *B. breve* (complete and incomplete) genome sequences. In this way, a comparable number of genes was obtained for each genome, with an average value of 1817 Open Reading Frames (ORFs) per genome (Table 2). Notably, a (BLAST-based) functional *in silico* prediction could be made for 74% of the identified ORFs, while the remaning 26% were predicted to encode hypothetical proteins.

**Table 2:** **General features of eight complete genomes of** ***Bifidobacterium breve***

FEATURE	***B. breve*** UCC2003	***B. breve*** S27	***B. breve*** 689b	***B. breve*** NCFB 2258	***B. breve*** JCM 7017	***B. breve*** JCM 7019	***B. breve*** 12L	***B. breve*** ACS-071-V-Sch8b	Average value
**Genome length (bp)**	2,422,684	2,294,458	2,331,707	2,315,904	2,288919	2,359,009	2,244,624	2,327,492	2,323,100
**Number of genes**	1854	1748	1821	1834	1770	1915	1765	1826	1817
**tRNA**	54	51	52	52	52	55	52	53	53
**rRNA**	2	3	2	2	2	2	2	2	2
**Hypothetical proteins**	27%	27%	28%	26%	24%	27%	27%	20%	26%
**Genes with assigned function**	73%	73%	72%	74%	76%	73%	73%	80%	74%
**IS elements/ transposases**	49	25	32	36	38	54	40	12	36
**Plasmid**	-	-	-	1	-	-	-	-	1
**Prophage/Integrated episome**	1 (remnant)	1	1 (remnant)	-	-	1	-	-	1
**CRISPR**	1	1	-	1	1	1	-	1	1

As displayed in Table 2, all fully sequenced genomes were observed and experimentally verified to encompass two identical rRNA loci located at non-adjacent positions in the genome with the exception of *B. breve* S27 which contains three of such loci; an average of 53 dispersed tRNA genes were noted per *B. breve* genome.

As previously observed in other bifidobacterial genomes [8, 13–15], the ATG sequence appears to be the preferred start codon (87%), while GTG, TTG and CTG seem to be less frequently used, with a calculated frequency percentage of 9.53%, 3.24% and 0.08%, respectively.

**Figure 1:**
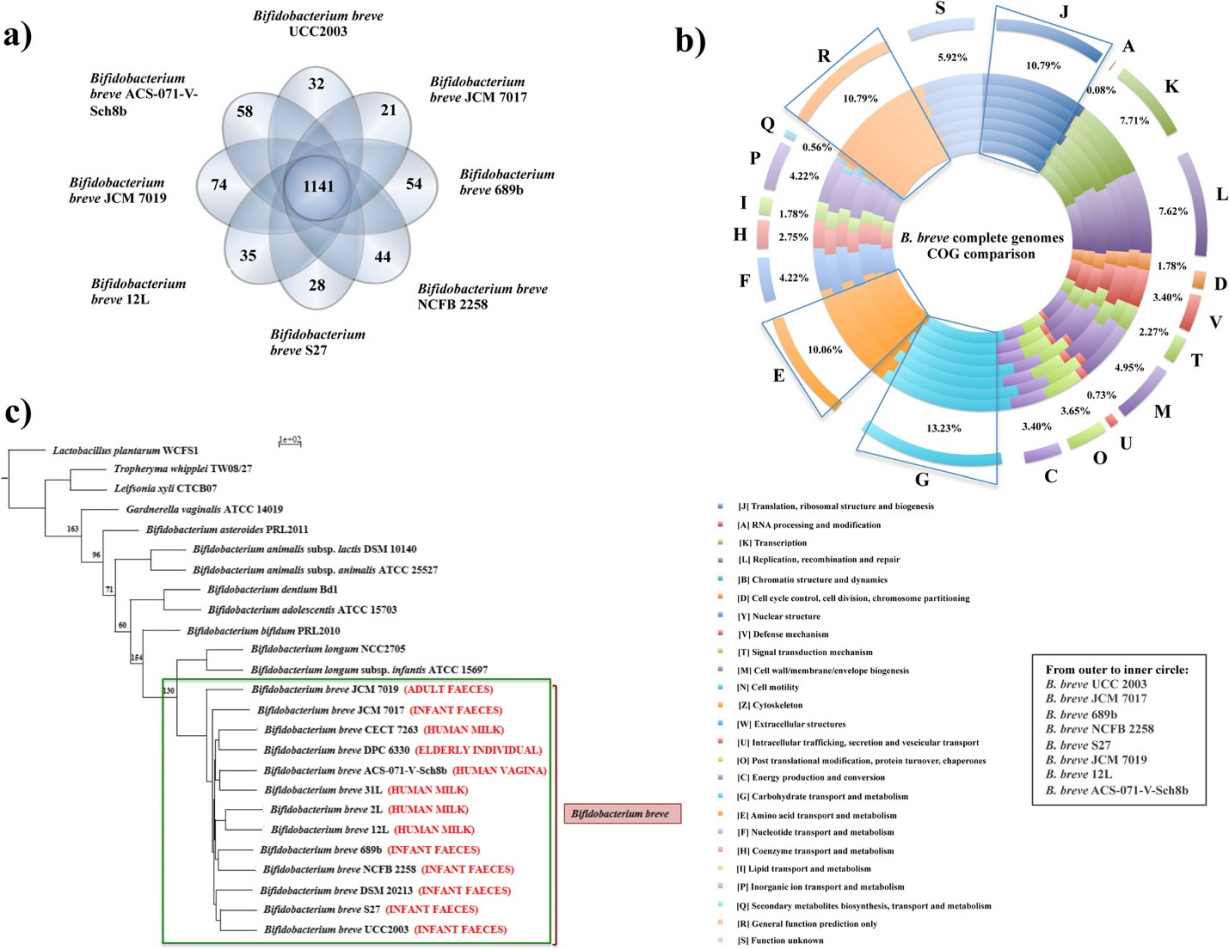
**Comparative genomics of fully sequenced** ***B. breve*** **genomes and phylogenetic supertree. a)** Venn diagram representing the orthologous and unique gene families as based on BLASTP comparison (E-value cut-off of 0.0001) and MCL clustering algorithm analyses. **b)** Cluster of Orthologues (COG) classification of the 1141 families of orthologues. For each COG entry the average percentage of hits among *B. breve* has been indicated. The most abundant families have also been indicated and they are assigned to housekeeping functions. From outer to inner circle: *B. breve* UCC2003, *B. breve* JCM 7017, *B. breve* 689b, *B. breve* NCFB 2258, *B. breve* S27, *B. breve* JCM 7019, *B. breve* 12L, *B. breve* ACS-071-V-Sch8b. **c)** Phylogenetic supertree showing the relationship between thirteen *B. breve* strains (*B. breve* UCC2003*, B. breve* 689b*, B. breve* 12L*, B. breve* NCFB 2258*, B. breve* S27*, B. breve* JCM 7017*, B. breve* JCM 7019*, B. breve* ACS-071-V-Sch8b*, B. breve* 2L*, B. breve* 31L*, B. breve* CECT 7263*, B. breve* DPC 6330*, B. breve* DSM 20213), *B. longum* subsp. *longum* NCC2705, *B. longum* subsp. *infantis* ATCC 15697, *B. bifidum* PRL2010, *B. adolescentis* ATCC 15703, *B. dentium* Bd1, *B. animalis* subsp. *animalis* ATCC 25527, *B. animalis* subsp. *lactis* DSM 10140 and *B. asteroides* PRL2011), three actinobacteria (*G. vaginalis* ATCC 14019, *L. xyli* subsp. *xyli* CTCB07 and *T. whipplei* TW08/27) and a single member of the *Firmicutes* as an outlier (*Lb. plantarum* WCFS1).

BLASTP comparisons performed between all ORFs identified on the eight fully sequenced genomes (as it produces a better definition of the variable regions; Figure 1) revealed the presence of 1141 orthologues (i.e. homologous genes, which are present in single copy, that are considered to have evolved vertically from a single ancestral gene) in addition to 924 gene families whose presence was observed in some but not all of the eight investigated *B. breve* members (also named shared genes). The comparative analyses of the eight fully sequenced *B. breve* genomes also allowed the identification of an average of 53 unique genes per genome, mostly representing hypothetical functions or mobile elements (Figure 1, panel a). The Cluster of Orthologous Group (COG) classification performed for the identified orthologous genes showed that the majority of these genes are predicted to be involved in various housekeeping functions, especially those related to carbohydrate and amino acid metabolism, and associated transport activities, in agreement with the general features previously observed for bifidobacteria (Figure 1, panel b) [13–16].

### The predicted mobilome of *B. breve*species

All complete genome sequences were investigated for the presence of mobile elements such as IS elements and genes specifying transposases, and this analysis revealed that the *B. breve* JCM 7019 genome contains the largest number (i.e. 54) of such mobile elements, while the *B. breve* ACS-071-V-Sch8b genome encompasses just 12 IS elements and transposase-encoding genes. The IS classification according to the ISFinder database [17] also showed that IS30 is the most frequently occurring insertion family in *B. breve*.

The complete chromosomes were also examined for the presence of prophages and plasmids. The prophage-like DNA element Bbr-1 of *B. breve* UCC2003 was previously analysed and represents a likely prophage-remnant [18]. Notably, in our analysis, we identified two other prophage like-elements (Additional file 1: Figure S1), 689b-1 in *B. breve* 689b (represented by the DNA region occupied by locus tags B689b_0284 through to B689b_0311), which appears to be incomplete, and 7019-1 in *B. breve* JCM 7019 (encompassing locus tags B7019_0905 through to B7019_1003), which appears to represent a complete prophage. Notably, the *B. breve* S27 genome appears to contain an integrated episome S27-1 (encompassing locus tags BS27_1090 through to BS27_1136), which is predicted to specify several hypothetical proteins, an integrase (BS27_1090), a DNA transfer protein (BS27_1114), and a cell wall anchor domain protein (BS27_1125). An extra-chromosomal (plasmid) sequence was confirmed to be present in *B. breve* NCFB 2258, and this plasmid is 100% identical to the previously published pCIBb1 [19] in full length BLASTN alignment.

### Whole-genome alignments and phylogenetic analysis

The eight fully sequenced *B. breve* genomes were also aligned using *B. breve* UCC2003 as the reference chromosome. The observed degree of alignment as displayed in a dot-plot exhibited a near-continuous straight line, indicating that all these genomes are highly syntenic, with the only exception of apparent inversions in the middle of the genome sequences of *B. breve* ACS-071-V-Sch8b (1126 Kb, corresponding to *B. breve* UCC2003 genome coordinates 611,964 - 1,653,404) and *B. breve* JCM 7017 (169 Kb, corresponding to UCC2003 genome coordinates 1,181,452 – 1,350,961) (Additional file 2: Figure S2). In the case of *B. breve* JCM 7017 we confirmed this genomic inversion by PCR, demonstrating that this was not due to an assembly error (data not shown). Furthermore, analysis of the DNA that directly flanks these two inversions revealed the presence of sequences specifying mobile elements/transposases, which may have acted as mechanistic drivers for this genomic reshuffling through homologous recombination [20]. *B. breve* ACS-071-V-Sch8b contains truncated integrases and transposases flanking the inversion (HMPREF9228_0467-69 and HMPREF9228_1435-38, respectively), while the *B. breve* JCM 7017 genome contains an hypothetical protein and a putative conjugative transposon at the left end of the inversion and sequences encoding a replication initiation factor, excisionase and integrase at the other inversion end (B7017_0896-97 and B7017_1053-55, respectively). A BLAST alignment performed on the above mentioned genes for *B. breve* JCM 7017 revealed high identity (88–100% in BLASTP alignment) with mobile elements found in *Clostridium difficile* 630 [21], which suggests their acquisition by means of horizontal gene transfer (HGT).

In order to investigate the phylogenetic relationship between *B. breve* and other bifidobacteria, a phylogenetic supertree was computed based on 165 orthologues, selected on the basis of the comparison of the thirteen *B. breve* genomes (see above), other sequenced *Bifidobacterium* species (*B. longum* subsp. *longum* NCC2705, *B. longum* subsp. *infantis* ATCC 15697, *B. bifidum* PRL2010, *B. adolescentis* ATCC 15703, *B. dentium* Bd1, *B. animalis* subsp. *animalis* ATCC 25527, *B. animalis* subsp. *lactis* DSM 10140 and *Bifidobacterium asteroides* PRL2011), and three additional actinobacterial genomes, (i.e. *Gardenerella vaginalis* ATCC 14019, *Leifsonia xyli* subsp. *xyli* CTCB07 and *Tropheryma whipplei* TW08/27), combined with a member of *Firmicutes* as a representative outgroup (*Lactobacillus plantarum* WCFS1). As shown in the resulting consensus tree (Figure 1, panel c) all thirteen *B. breve* members fall into the *B. longum* phylogenetic group, which is consistent with a previous assignment based on a multilocus approach [22]. As shown in a previous study the colonization of the infant gut by representatives of *B. breve* and *B. longum* occurs immediately after birth, with a correlation being observed between strains present in mother and progeny, thus suggesting that such bifidobacteria are transmitted from mother to child during vaginal delivery and/or breast feeding [5, 6]. The strains analyzed in this study possess very similar ecological origins and it was therefore not surprising that no clear separation of these strains was observed within the tree. However, *B. breve* JCM 7019, an isolate from adult faeces, clustered in a separate branch, while the *B. breve* milk isolates also cluster together. Additionally, most of the infant stool isolates were shown to cluster in the same group at the bottom of the tree.

### *B. breve*core and dispensable genome

Comparative genomic analysis based on BLASTP comparisons and MCL clustering algorithm between the eight complete *B. breve* genomes (see Methods) allowed the definition of a set of 1323 gene families, representing the core genome for the *B. breve* species, defined as a pool of gene families that is present in all of the considered genomes [10, 11], and representing the 1141 orthologues mentioned above plus an additional 182 paralogues. Inspection of corresponding COG assignments (Additional file 3: Figure S3) revealed that many components of this core genome represent functions related to cellular housekeeping. It is also worth mentioning that this set of core families is composed of common functions which can be present in single copy (named orthologues and including a large proportion of the identified housekeeping genes), but also functions present in multiple copy (also named paralogues, of which ATP Binding Cassette (ABC)-type transporters represent a typical example). Variability among the *B. breve* genomes is due to a specific set of functions also called dispensable genes which are present in more than one of the examined *B. breve* genomes, yet not present in all, as well as genes that are specific for just one strain [10, 11]. Our analysis revealed a total of 924 families of variable genes, 426 of which are classified as unique. Of these 924 gene families, 49% encode hypothetical proteins, while the remainder is assigned to more informative features, such as genes predicted to encode proteins involved in capsular exopolysaccharide (EPS) synthesis, in phage resistance (CRISPR locus and R/M systems), in the production of sortase-dependent pili, and in carbohydrate metabolism, including various carbohydrate transporters (Additional file 4: Table S1). Notably, our *in silico* data corroborate and extend previously published CGH analyses, that had been performed to explore the genomic diversity of *B. breve*[8].

Furthermore, the total gene pool (ORFeome) extracted from the eight *B. breve* complete genomes was compared with that of six complete and publicly available chromosome sequences of *B. longum* subsp. *longum* (*B. longum* subsp. *longum* NCC2705, *B. longum* subsp. *longum* DJO10A, *B. longum* subsp. *longum* JCM 1217, *B. longum* subsp. *longum* ATCC 15697, *B. longum* subsp. *longum* 157F, *B. longum* subsp. *longum* BBMN68), which is phylogenetically the closest related taxon to *B. breve*[22]. This comparison showed that 564 gene families (Additional file 5: Table S2) are specifically present in *B. breve*, yet absent in *B. longum* subsp. *longum*. Of these 564 gene families, approximately 50% encode unknown or hypothetical functions, while the other 50% represent functions similar to the ones observed in the variable regions of *B. breve* (i.e. glycosyl hydrolases, ABC transporters, CRISPR genes and mobile elements). The analysis also showed that 581 genes families are specifically present in *B. longum* subsp. *longum*, yet absent in *B. breve*, where approximately 68% are coding unknown or hypothetical functions, while the remaining 32% specify mobile elements, ABC transporters and glycosyl hydrolases (data not shown).

### Variability among *B. breve*genomes

**Figure 2:**
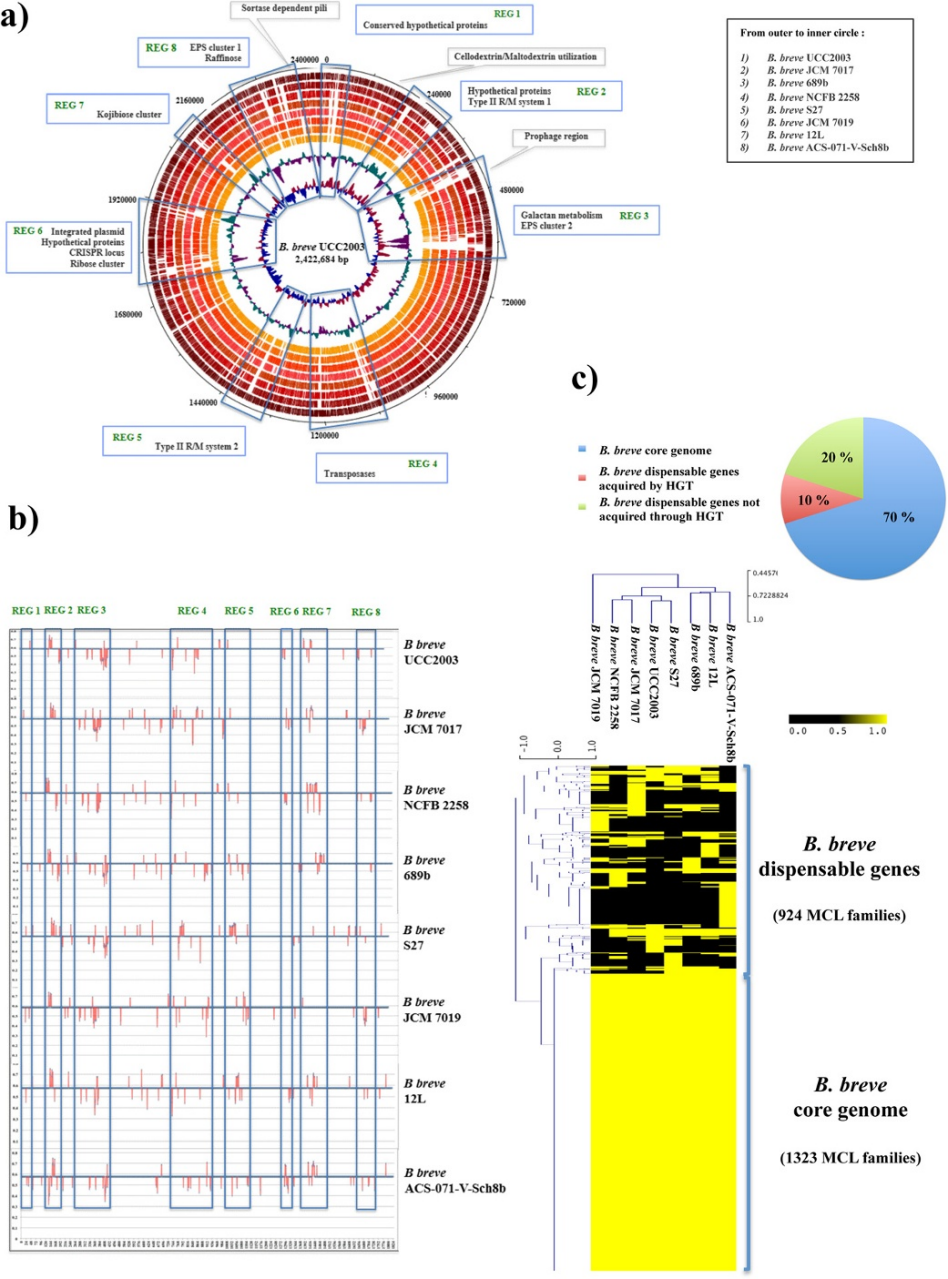
**Regions of variability among the** ***B. breve*** **genomes. a)** BLAST-based genome atlas showing the presence of each ORF from *B. breve* UCC2003 and the other *B. breve* complete representatives. From outer to inner circle: *B. breve* UCC2003, *B. breve* JCM 7017, *B. breve* 689b, *B. breve* NCFB 2258, *B. breve* S27, *B. breve* JCM 7019, *B. breve* 12L, *B. breve* ACS-071-V-Sch8b. **b)** Alignment showing in red the ORFs with significant G+C mol% in *B. breve* (*B. breve* UCC2003, *B. breve* 689b, *B. breve* 12L, *B. breve* NCFB 2258, *B. breve* S27, *B. breve* JCM 7017, *B. breve* JCM 7019, *B. breve* ACS-071-V-Sch8b); highlighted in grey are regions of variability (REG1-8) that were identified based on gene presence/absence and G+C mol% deviation in each strain. **c)** Hierarchical clustering heatmap representing the variability of *B. breve* in terms of presence/absence of genes for eight complete genomes of *B. breve* with associated percentage of core and variable gene families represented as pie chart.

In order to determine the presence of regions containing clusters of genes putatively acquired by HGT (Horizontal Gene Transfer) in *B. breve*, the G+C mol% for each ORF was calculated and only genes with a significantly different G+C content (i.e. higher that 68% or lower than 49%) were plotted (Figure 2, panel b). This analysis established that 10% of the total of gene families present in *B. breve* displays a deviating G+C content (Figure 2, panel c), the majority of which (85%) are mapped in eight variable genomic regions (indicated as REG1-8 in Figure 2, panel b) that were concordant with the regions of variability identified by a BLAST-based comparative analysis (Figure 2, panel a) and the variable regions previously detected by CGH [8]*.* As shown by the outcomes of a hierarchical clustering analysis that was performed to scrutinize the presence or absence of genes in the eight complete *B. breve* representatives, the overall of variability accounts for approximately 30% of the total of genes predicted for this species (a third of which is predicted to be acquired by HGT), while the remaining 70% represents the core genes (Figure 2, panel c).

These variable regions were shown to include the EPS cluster 2 (Bbr_0430-74, REG 3) containing two opposite orientated operons (*eps1* from Bbr_0441 to Bbr_0434 and the *eps2* from Bbr_0442 to Bbr_0451 [23], type II R/M systems 1–3, Bbr_0214-16 and Bbr_1118-21 [24], REG 2 and REG 5, respectively), conjugative transposon, excisionase and integrase of *B. breve* JCM 7017 (B7017_0896-97 and B7017_1053-55, REG 4), a CRISPR locus (Bbr_1405-11, REG 6), pilus-encoding genes (Bbr_1887-89), clusters encoding enzymes involved in the metabolism of carbohydrates (REG 7–8) and hypothetical proteins (REG 1) (Figure 2, panel a).

Comparative analysis conducted on the eight fully sequenced *B. breve* chromosomes revealed the presence of an apparently intact EPS cluster 2 in *B. breve* JCM 7017, *B. breve* JCM 7019, *B. breve* 689b and *B. breve* S27, positioned in an identical genomic location as *B. breve* UCC2003 with genes ranging between 100–50% of similarity in BLASTP alignments. In contrast, *B. breve* ACS-071-V-Sch8b, *B. breve* NCFB 2258, and *B. breve* 12L appear to only contain a remnant EPS biosynthesis cluster, where the gene encoding a presumed priming glycosyl transferase is present, while many other genes related to EPS biosynthesis are absent (Table 3; Additional file 6: Figure S5, panel a). Interestingly, two EPS clusters were observed in the draft genome *B. breve* 31L (B31L_0002-0010 and B31L_1353-84), one of which containing several transposases and located in the same genomic position as the EPS cluster 2 of *B. breve* UCC2003. A second putative exopolysaccharide biosynthesis cluster named EPS cluster 1 (Bbr_1786-1801, unpublished data), was also shown to be present in the other analysed *B. breve* genomes and it appears to be more conserved than EPS cluster 2 among the strains analyzed (Additional file 6: Figure S5, panel b). A CRISPR locus, previously described for *B. breve* UCC2003 [8], was found in the chromosome of *B. breve* S27, *B. breve* ACS-071-V-Sch8b, *B. breve* NCFB 2258, *B. breve* JCM 7017 and *B. breve* JCM 7019, where variability was observed in the spacer region of each strain-specific CRISPR (Table 3). In contrast, a CRISPR locus appears to be absent from the chromosomes of *B. breve* 689b and the *B. breve* milk isolates (*B. breve* 12L, *B. breve* 2L, *B. breve* 31L).

**Table 3:** ***Bifidobacterium breve*** **variable regions**

Variable regions	***B. breve*** UCC2003	***B. breve*** S27	***B. breve*** 689b	***B. breve*** NCFB 2258	***B. breve*** JCM 7017	***B. breve*** JCM 7019	***B. breve*** 12L	***B. breve*** ACS-071-V-Sch8b
**EPS cluster 2**	Bbr_0430-0474	BS27_0430-0465	B689b_0425-0453	B2258_0400-0427	B7017_0382-0428	B7019_0391-0428	(B12L_0366-0393)	(HMPREF9228_0447-58)
**EPS cluster 1**	Bbr_1786-1803	BS27_1781-1804	B689b_1819-1843	B2258_1811-1826	B7017_1982-2015	B7019_1956-1987	B12L_1718-1745	HMPREF9228_1868-92
**CRISPR**	Bbr_1405-1411	BS27_1428-1434	-	B2258_1384-1390	B7017_1609-1615	B7019_1592-1598	-	HMPREF9228_1444-1451
**R/M system 1**	Bbr_0214-0216	BS27_0382-0383	B689b_1504-1505	B2258_0195-0196	B7017_0735-0736	B7019_0015-0016	B12L_1341-1346	HMPREF9228_1774-75
**R/M system 2**	Bbr_1118-1119	-	-	B2258_0357-0358	B7017_1663-1664	B7019_0077-0079	-	-
**R/M system 3**	Bbr_1120-1121	-	-	-	-	B7019_0197-0198	-	-
***pil1***	Bbr_0113-0115	BS27_0127-29	B689b_0101-0103	B2258_0100-0102	B7017_0130-0132	B7019_0110-0112	B12L_0104-0106	HMPREF9228_0113-15
***pil2***	Bbr_0365-0366	BS27_0354-55	B689b_0357-0358	B2258_0329-0330	B7017_0315-0316	B7019_0326-0327	B12L_0301-0302	HMPREF9228_0369-70
***pil3***	Bbr_1887-1889	-	-	B2258_1894-1896	B7017_2091-2093	-	-	-

A varying number of predicted type II DNA R/M systems were identified in each of the eight completed *B. breve* genomes. The chromosomes of *B. breve* UCC2003 and *B. breve* JCM 7019 are each predicted to encode three R/M systems, while the chromosomes of *B. breve* JCM 2258 and *B. breve* JCM 7017 each encompass two such systems, and the chromosomes of the remaining strains *B. breve* 12L, *B. breve* 689b, *B. breve* ACS-071-V-Sch8b, *B. breve* S27 are each predicted to contain a single R/M system (Table 3).

Regarding genes that encode (predicted) adhesion factors, a type IVb tight adherence (*tad*) locus was previously characterized in *B. breve* UCC2003 [8] and its presence was also observed in all other *B. breve* strains with an high degree of similarity (100–98% in BLASTP alignment; Additional file 7: Figure S4, panel a). In contrast to the Tad-like genes, the analyzed *B. breve* genomes were shown to contain a varying number of sortase-dependent pilus-encoding loci: *B. breve* UCC2003, *B. breve* NCFB 2258 and *B. breve* JCM 7017 contain three sortase-dependent pili loci (designated *pil1*, *pil2* and *pil3*), while *B. breve* JCM 7019, *B. breve* 12L, *B. breve* 689b, *B. breve* ACS-071-V-Sch8b, *B. breve* S27 only contain 2 (*pil1* and *pil2*; Table 3) (Additional file 7: Figure S4, panel b). In most cases (with the only exception of *B. breve* 12L where the clusters also appear to lack a dedicated sortase-encoding gene), an apparent frameshift within a 10–11 guanine nucleotide stretch in the first surface protein-encoding gene of *pil1* and *pil3* was present, a phenomenon previously observed for *B. breve* UCC2003 [8], as well as for *B. bifidum* PRL2010 [9, 25].

### Pan-genome analysis

**Figure 3:**
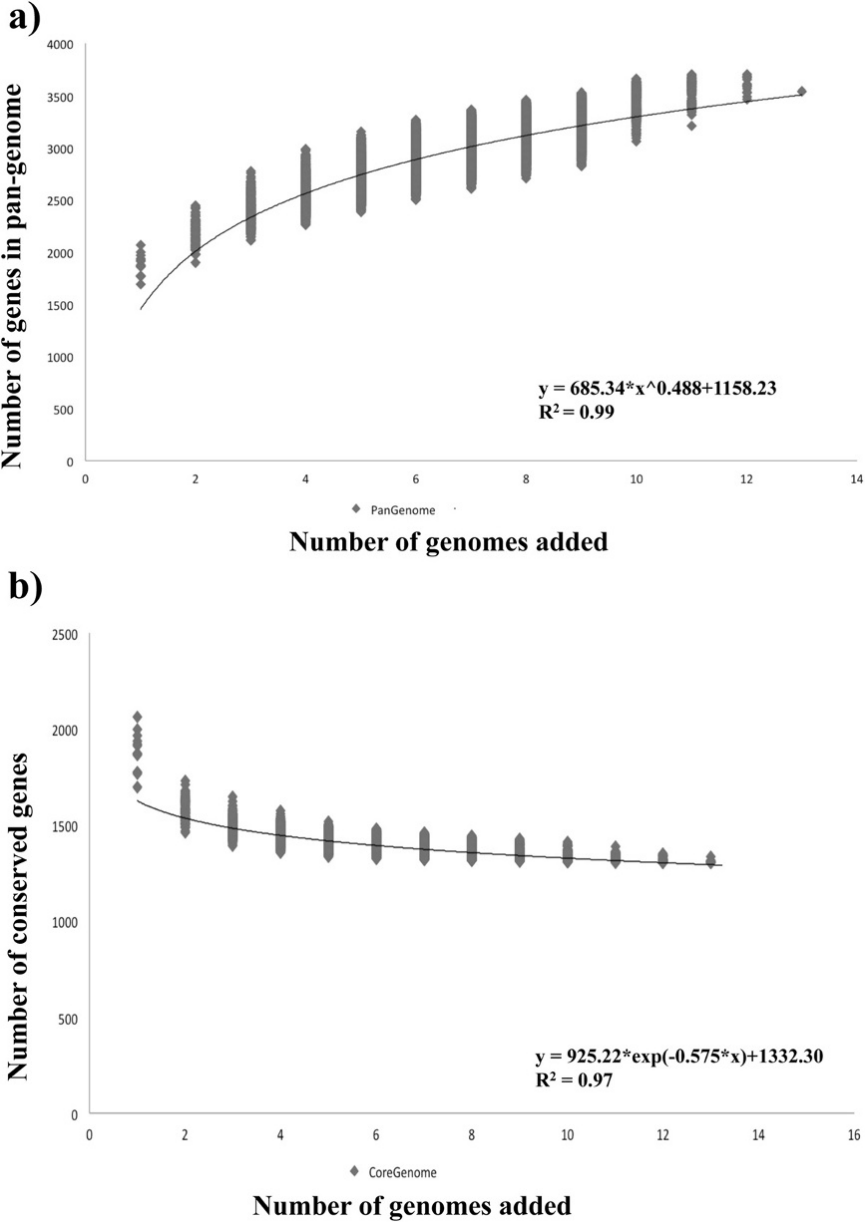
**Pan-genome and core-genome of** ***B. breve.*** **a)** Accumulated number of new genes in the *B. breve* pan-genome plotted against the number of genomes added. The deduced mathematical function is also indicated. **b)** Accumulated number of genes attributed to the core-genome plotted against the number of added genomes. The deduced mathematical function is also reported.

In order to estimate the total number of different genes present in representatives of the *B. breve* species, and the expected number of new genes for every inclusion of an additional *B. breve* genome, we applied a pan-genome analysis pipeline named PGAP v1.0 [26]. A total number of thirteen *B. breve* genomes (both complete and incomplete genome sequences) were included in this analysis, and from the results as displayed in Figure 3 the pan-genome curve displays an asymptotic trend, growing with an average rate of 196 genes per genome in the first nine iterations, after which the number of new genes rapidly decreases and where the resulting curve leads to a value of 3667 genes. This suggests that following the inclusion of nine genomes, the incorporation of additional genomes only leads to a minor increase in the pan genome size. In fact, *in silico* analysis of such ‘new’ genes show that they mostly encode small and hypothetical proteins. Similar results were achieved using the core-genome function, where the asymptotic trend is even more evident and after the 9th genome iteration the total number of genes in the core genome stabilizes to a value of 1307, which is comparable with the number of gene families extracted in our comparative analysis (see above). Both trends observed in the pan-genome and core-genome functions indicate that *B. breve* displays an essentially closed pan-genome, and that the number of genomes analyzed here is sufficient to describe the complete gene repertoire of this bifidobacterial species.

### Carbohydrate utilization

As from the previous CGH work performed on 18 *B. breve* isolates [8], genetic variability among the analysed representatives of this group was observed for genes previously characterized as being involved in the utilization of the carbohydrates ribose [27], sucrose [28] and raffinose [29], as well as the plant-derived polysaccharides starch [30], galactan [31] and cellodextrin [32].

Interestingly, in all analysed strains, genes are present that are predicted to encode enzymes involved in the uptake and utilization of host-derived mono/oligosaccharides, in particular mucin and Human Milk Oligosaccharides (HMOs). Examples of this include gene clusters predicted to be involved in the metabolism of sialic acid (Bbr_0160-73 and Bbr_1247), lacto-*N*-biose through a Leloir-like metabolic pathway [33] (Bbr_1587, Bbr_0491, Bbr_1884 and Bbr_1585), fucose (Bbr_1740-42) and *N*-linked glycans [34] (Bbr_1141-50). Although *B. breve* is not known to be able to grow on mucin or HMOs [35, 36], host-derived mono/oligosaccharides may become available through hydrolytic activities of other (bifido) bacteria present in the gut (e.g. *B. bifidum* PRL2010 [14] and *B. longum* subsp. *infantis*[37]), allowing *B. breve* strains to utilize such liberated carbohydrates through a phenomenon called cross-feeding [38].

**Figure 4:**
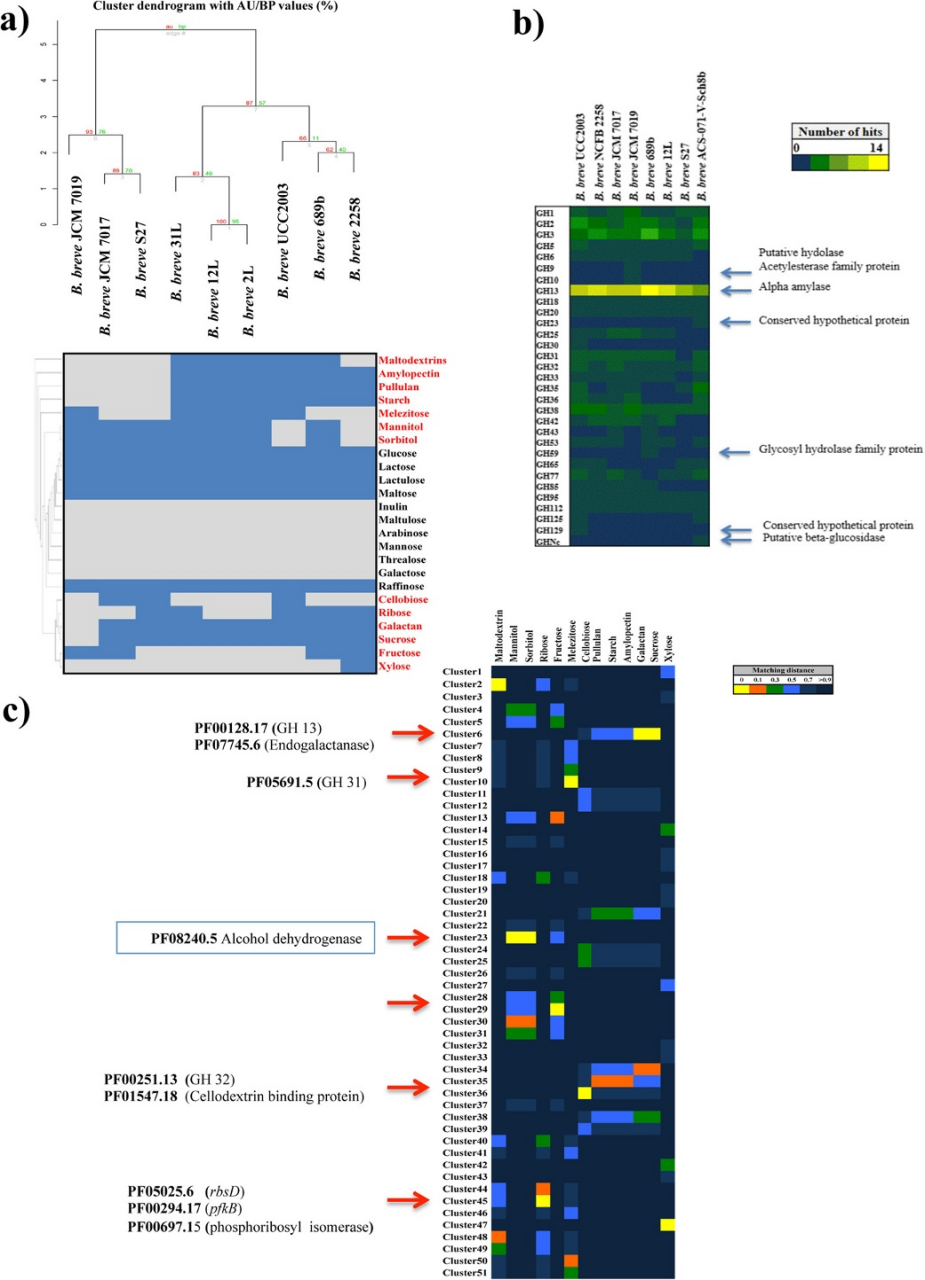
***B. breve*** **carbohydrate profiling. a)** Hierarchical clustering analysis performed on the phenotype observed in *B. breve* UCC2003, *B. breve* NCFB 2258, *B. breve* 12L, *B. breve* 2L, *B. breve* 31L, *B. breve* 689b, *B. breve* JCM 7017 and *B. breve* JCM 7019, *B. breve* S27 tested for growing on 24 sugars. **b)** The *in silico* prediction and numerical presence of all identified GH family members according to the Cazy classification in the eight fully sequenced *B. breve* strains. **c)** Heatmap showing the *in silico* gene-trait matching exercise performed on the 51 clusters that were derived from the hierarchical clustering analysis. The relative matching distance of each cluster is indicated with colour gradient and the main results are highlighted.

In order to extend our knowledge on carbohydrate fermentation capabilities of *B. breve*, an *in silico* prediction of all glycosyl hydrolases (GHs) was first performed on the eight complete *B. breve* genomes. This analysis essentially confirmed an abundance of members of GH family 13 (α-amylase function), which was previously defined as a distinctive characteristic of *B. breve*[30]. It also highlighted the presence of a small number of GHs that appear to be present in just a single member of *B. breve* (GH9, GH10, GH23, GH59, GH129 and GHNc [39]; Figure 4, panel b), and their annotation suggests that they may encode novel hydrolytic activities.

Comparing carbohydrate fermentation profiles of nine *B. breve* strains (*B. breve* UCC2003, *B. breve* 689b, *B. breve* 12L, *B. breve* 2 L, *B. breve* 31 L, *B. breve* 2258, *B. breve* JCM 7017, *B. breve* JCM 7019 and *B. breve* S27) revealed that all strains are able to ferment a common set of sugars, such as glucose, lactose, lactulose, maltose and raffinose. In contrast, fermentation capabilities for the other sugars such as galactan, sucrose, pullulan, amylopectin, starch, maltodextrins, sorbitol, mannitol, fructose, melezitose, cellobiose, xylose and ribose, were shown to be variable among the strains tested. None of the *B. breve* strains assayed here was shown to be capable of utilizing inulin, arabinose, maltulose, mannose, trehalose and galactose (Figure 4, panel a).

In bifidobacteria, genes involved in the utilization of a given sugar are frequently organized in gene clusters containing genes that encode one or more specific GHs and associated transport system, and are frequently placed under the transcriptional control of a LacI-type regulator specified by a gene that is also located adjacent to or within such a gene cluster [27–32].

A gene-trait matching analysis performed on these *B. breve* strains, based on the association between the 51 presence/absence clusters of genes (named Cluster1 through to Cluster51) obtained by hierarchical clustering (HCL) analysis and growth/no growth phenotype, allowed an *in silico* assessment of the role of certain genes associated with carbohydrate metabolism and carried by the *B. breve* chromosomes sequenced here, several of which had previously been characterized in *B. breve* UCC2003 [27–32]. This analysis was carried out on the sugars that generated differential carbohydrate profiles among the tested *B. breve* strains (Figure 4, panel a), and allowed the identification of 34 genes that correspond with a strain’s ability to grow on ribose, galactan, sucrose, melezitose, cellobiose, mannitol and sorbitol (Table 4).

**Table 4:** **Gene-trait matching with functions resulting from hierarchical clustering analysis**

Carbohydrate	HCL cluster	Functions
**Ribose**	**Cluster45**	**Ribose transport system permease protein rbsD**
		Conserved hypothetical membrane spanning protein
		**pfkB family carbohydrate kinase**
		NADH-dependent butanol dehydrogenase 1
		Phosphoglycolate phosphatase
		Inosine-uridine preferring nucleoside hydrolase
		pfkB family carbohydrate kinase
		N-(5’-phosphoribosyl) anthranilate isomerase
		Cobalt transport protein cbiQ
		Conserved hypothetical membrane spanning protein
		**Ribokinase**
**Galactan/Sucrose**	**Cluster6**	**Amylosucrase or alpha-glucosidase**
		**Glycosyl hydrolases family 53, Endogalactanase, galactan metabolism**
		Conserved hypothetical protein, PhoU-like domain
		Transporter
		Transcriptional regulator
		Narrowly conserved hypothetical membrane spanning protein, MFS superfamily
		Narrowly conserved hypothetical membrane spanning protein
		Conserved hypothetical membrane spanning protein
		Transporter
**Melezitose**	**Cluster10**	**Raffinose synthase or seed imbibition protein Sip1/Alpha-galactosidase**
**Cellobiose**	**Cluster36**	Glycosyl hydrolases family 32, Beta-fructosidase or sucrose-6-phosphate hydrolase
		**Cellodextrin binding protein**
**Mannitol/sorbitol**	**Cluster23**	AraC family transcriptional regulator
		**Alcohol dehydrogenase**
		**Putative glyoxalase family protein**
		**Alcohol dehydrogenase**
		Sugar isomerase (SIS)
		Putative ABC transporter, permease protein
		ABC transporter, permease protein
		ABC transporter, permease protein
		Xylulokinase
		Putative ROK family protein

**Figure 5:**
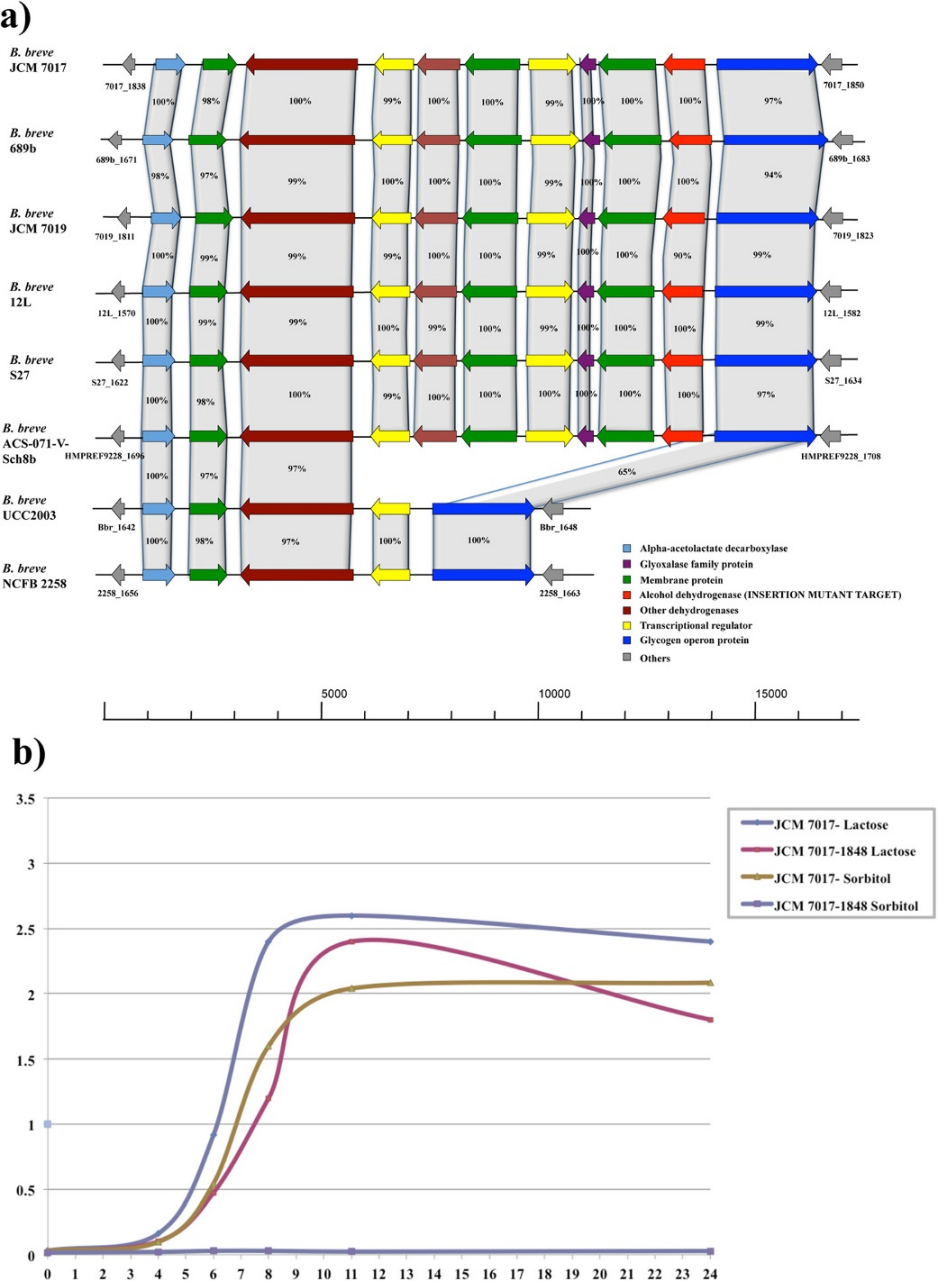
**Sorbitol gene cluster in** ***B. breve*** **and insertion mutant growth curve. a)** Locus map showing the comparative analysis of the gene cluster putatively involved in the utilization of the sugar alcohol sorbitol in certain *B. breve* chromosomes*.* All genes are coloured coded based on their function. The percentage of similarity based on BLASTP alignment and the alcohol dehydrogenase-encoding gene targeted for the genetic insertion experiment are indicated. **b)** Diagram showing the growth curves of *B. breve* JCM 7017 wild-type and *B. breve* JCM 7017–1848 insertion mutant on Rogosa modified MRS (mMRS) with the addition of lactose and sorbitol 0.5% over 24 hours.

For example, growth on ribose was shown to correspond with Cluster45, which contains genes that have previously been described to be required for ribose utilization [27], such as *rbsD*, encoding a component of the presumed ribose transport system, and *rbsK*, encoding a ribokinase. The obtained strain-specific growth profile distribution on galactan corresponds with Cluster6, which encompasses a gene specifying an endogalactanase (Bbr_0422), which has been previously shown to be involved in galactan metabolism in *B. breve* UCC2003 [31]. Also, strain-specific growth on melezitose corresponds to Cluster10, which includes a gene encoding an alpha-galactosidase (Bbr_1856) shown to be required for the utilization of this carbohydrate [40, 41]. Furthermore, this analysis allowed the identification of a gene cluster in *B. breve* JCM 7017 (ORFs B7017_1846-1848), which is predicted to encode elements for the regulation, transport and metabolism of the sugar alcohol sorbitol by some members of the *B. breve* taxon (Figure 5, panel a). Notably, this locus encompasses an alcohol dehydrogenase-encoding gene (B7017_1848), which was targeted for insertion mutagenesis, and followed by a gene (B7010_1847) predicted to specify an ABC transporter, and presumed to be responsible for internalization of this carbohydrate, and gene (B7017_1845) encoding a predicted a transcriptional regulator. As compared to the wild type *B. breve* JCM 7017, the insertion mutant strain, designated here as *B. breve* JCM 7017–1848, was incapable of using sorbitol as a sole carbohydrate source, thereby confirming the role of this gene cluster in sorbitol metabolism (Figure 5, panel b).

## Conclusions

Genome sequencing of eight *B. breve* strains and comparative analysis of these genomes, combined with five additional, publicly available *B. breve* genomes, allowed the description of the pan-genome of the *B. breve* species, which was shown to follow an essentially closed trend. As pan-genomic analysis was only recently introduced for the description of bacterial species [10], its application is still somewhat controversial and subject to scientific scrutiny. One clear limitation of pan-genome analysis is the difficulty of assessing whether a pan-genome is really closed or not, given the dynamic nature of a given bacterial population and its associated tendency to evolve and exchange genetic material. In general it can be said that a closed pan-genome implies that the gene exchange within a species is low and this certainly seems to be the case for the thirteen *B. breve* genomes analyzed here.

Moreover, the *in silico* prediction of ORFs with a deviating G+C mol% content coupled with a comparative genomics analysis, allowed the identification of eight genomic regions of variability in the *B. breve* pangenome representing approximately 30% of the total of gene content within the *B. breve* species, containing a large fraction of ORFs that have been acquired by HGT (which constitutes the 10% of the total of gene content in *B. breve*). Apart from hypothetical proteins and mobile elements, the gene functions contained within these variable regions are predicted to be required for environmental niche adaptation of this group. For a gut commensal the process of colonization involves cell-cell and cell-host interactions (involving for example genes that encode sortase-dependent pili for adhesion, biofilm formation and cell aggregation [8, 9]) and evasion of the host adaptive immune response [23] (requiring genes specifying the biosynthesis of an exopolysaccharide capsule), as well as metabolic flexibility to acquire energy from a variety of carbon sources independent of the age of the host (when the host shifts from a milk-only diet to a diversity of solid foods, thus explaining the predicted capacity of producing a wide variety of GHs). Furthermore, evolutionary pressures to resist invasion of foreign DNA (e.g. phages and plasmids) also appears to provide an explanation for the presence of CRISPR, CRISPR-associated genes, as well as R/M systems in the variable regions of *B. breve*.

The comparative genomics approach used in this study also facilitated the explanation of certain differences previously observed in the carbon sources utilization in some *B. breve* members and allowed the definition of a new cluster responsible for the fermentation of the sugar alcohol sorbitol.

For this reason the *in silico* analysis presented in this study represents a robust starting point for future functional genomics investigations focusing on (individual members of) this bifidobacterial species, in order to elucidate the spectrum of functions and mechanisms of interaction with the host environment to explain the presence of these bacteria in the human gut and the reported beneficial effects on their host.

## Methods

### Genome sequencing and data assembly

All genomes used in this study are human isolates of *B. breve*, of which *B. breve* UCC2003 was isolated and sequenced as part of a previous study [8], *B. breve* 12L, *B. breve* 2L and *B. breve* 31L, *B. breve* 689b, *B. breve* S27 were isolated from human milk and breast-fed infant feaces as previously described [42], while *B. breve* JCM 7017, *B. breve* JCM 7019, *B. breve* NCFB 2258 were obtained from the Japan Collection of Microorgnisms and National Collection of Food Bacteria, respectively). All genomes were sequenced using one or more Next Generation Sequencing (NGS) platforms. In order to construct an initial scaffold backbone, reads were first obtained using a 454 Roche genome sequencer FLX Titanium instrument employing a long-tag, paired-end library (average read length of 400 bp).

The genomes of *B. breve* 689b, *B. breve* 12L and *B. breve* S27 were sequenced using a Roche 454 FLX Titanium instrument by the commercial sequencing service providers Agencourt Bioscience (Beverly, MA) and Eurofins MWG Operon (Germany) and then assembled, after which remaining gaps were closed using Sanger sequencing of PCR products. The obtained consensus genome sequence consisted of an approximately 30-fold overall coverage, where any remaining low quality regions or other sequence conflicts were resolved using additional Sanger sequencing of PCR products. Assembly was performed using Newbler v2.6 (http://454.com/products/analysis-software/index.asp) and Gap4 (Staden package v1.6.0 (http://sourceforge.net/projects/staden/).

In the case of the genomes of *B. breve* NCFB 2258, *B. breve* JCM 7017 and *B. breve* JCM 7019, sequences were obtained using a combination of the afore mentioned 454 FLX Titanium and Illumina Hiseq 2000 sequencing platforms, both performed by Macrogen (Seoul, Republic of Korea) (and using a paired-end library with an average read length of 450 bp and 101 bp, respectively). The obtained sequences were assembled employing a hybrid assembly using a combination of Newbler v2.6 (http://454.com/) for long reads and Abyss v1.3.4 (http://www.bcgsc.ca/) for short reads, resulting in a 200-fold coverage. Any remaining gaps and quality issues were resolved using Sanger sequencing of PCR products.

Finally, the draft genome sequences of *B. breve* 2L and *B. breve* 31L were carried out by GenProbio Ltd. (http://genprobio.com) combining the output of two runs of Ion Torrent PGM (Life Technologies, Germany) following a previously described protocol [43], reaching a coverage of approximately 50-fold. The obtained raw data were assembled using MIRA v.3.9 (http://www.chevreux.org/projects_mira.html), applying default parameters recommended for Ion Torrent data processing. All reads were quality checked and trimmed in order to improve the assembly process; this quality check/trimming step was performed for both 454 FLX and Illumina data using NGSQCToolkit v.2.3 [44]. For Ion Torrent reads the pre-processing step was performed using a built-in function of the Mira assembler software (v3.9) (http://www.chevreux.org/).

### General features prediction

Open Reading Frame (ORF) prediction was performed with a combined approach of the predictor Prodigal v2.0 (http://prodigal.ornl.gov) and BLASTX v2.2.26 [45] alignment for all the genomes analysed in this study; identified ORFs were then automatically annotated on the basis of BLASTP v2.2.26 [45] analysis using *B. breve* UCC2003 as the reference genome (NCBI Reference Sequence: NC_020517.1). Functional assignment was performed and manually edited based on similarity searches against the non-redundant protein database curated by the National Centre for Biotechnology Information (ftp://ftp.ncbi.nih.gov/blast/db/).

Artemis v.14 (http://www.sanger.ac.uk/resources/software/artemis/) was used to combine and inspect the results of the ORF finder and the associated BLASTP [45] results, while this software tool was also used for manual editing, where necessary, of the start codon of a predicted gene. Where appropriate, annotations were further refined, verified or adjusted using information retrieved from alternative databases, e.g. Uniprot/EMBL (http://www.uniprot.org/), protein family (Pfam) (http://pfam.sanger.ac.uk) and COGs [46].

Transfer RNA genes were identified using tRNAscan-SE v1.4 [47] and ribosomal RNA genes were detected on the basis of Rnammer v1.2 [48] and BLASTN v2.2.26 [45] searches and annotated manually. Insertion sequence elements were identified and assigned using IS finder (https://www-is.biotoul.fr) and BLAST v2.2.26 [45] and annotated manually. Carbohydrate-active enzymes were identified based on similarity to the carbohydrate-active enzyme (CAZy) database entries [39], Enzyme Commission numbers (http://enzyme.expasy.org) and Pfam alignments (http://pfam.sanger.ac.uk), and this combined information was used for manual annotation purposes.

Deviations in G+C mol% were computed based on the ORFs nucleotide sequence using Geecee function from the EMBOSS v6.5.7 package [49].

All genome sequences were searched for the presence of Restriction/Modification systems using a BLASTP [45] alignment function of the REBASE database (http://rebase.neb.com/rebase/rebase.html) (cut-off E-value of 0.0001; and at least showing 30% of similarity of at least 80% of the sequence length).

### Comparative genomics

Whole-genome sequence alignments were performed at the DNA level using the software package MUMmer v3.0 [50]. Sequence comparisons at protein level were performed using an all-against-all, bi-directional BLAST alignment [45] (cut-off: E-value 0.0001, with at least 50% identity across at least 50% of either protein sequence), and the resulting output was then clustered into protein families sharing the same function using the Markov Cluster Algorithm (MCL) implemented in the mclblastline pipeline v12-0678 [51]. The obtained gene families were classified as belonging to either the core or to the dispensable genome based on their presence in either all strains or in a subset of the investigated strains, respectively. In the orthologues extraction an additional filter for paralogues was applied by selecting only those families that were shown to contain a single protein member for each genome. Proteins identified as belonging to the mobilome, such as IS elements or phages, were also discarded from this pool of genes and orthologues were then functionally classified using COG category assignments [46].

### Phylogenetic analysis

The supertree computation was performed from the alignment of a set of orthologous genes obtained from the same BLAST-based comparative approach as indicated above (Additional file 8: Table S3).

Each protein family was aligned using CLUSTAL_W v1.83 [52]. Phylogenetic trees were computed using the maximum-likelihood in PhyML v3.0 [53] and concatenated; the resulting consensus tree was computed using the Consense module from Phylip package v3.69 using the majority rule method (http://evolution.genetics.washington.edu/phylip.html) and phylogenetic data were submitted to TreeBASE database (http://treebase.org/treebase-web/home.html).

### Pangenome calculation

For the available *B. breve* genomes a pan-genome computation was calculated using the PGAP v1.0 [26], which performs this analysis according to the Heap’s law pan-genome model [10]; the ORF content of each genome is organized in functional gene clusters using the GF (Gene Family) method and a pan-genome profile was then built.

### Carbohydrates fermentation profiles

In order to investigate their carbohydrate-utilization capabilities, nine *B. breve* strains, which were available to us (*B. breve* UCC2003, *B. breve* 689b, *B. breve* 12L, *B. breve* 2L, *B. breve* 31L, *B. breve* 2258, *B. breve* JCM 7017, *B. breve* JCM 7019 and *B. breve* S27) were experimentally tested for growth on 24 different carbohydrates (glucose, lactose, lactulose, maltose, raffinose, galactan, sucrose, pullulan, amylopectin, starch, maltodextrin, sorbitol, mannitol, fructose, melezitose, cellobiose, inulin, arabinose, maltulose, mannose, trehalose, galactose, xylose and ribose). The seven *B. breve* strains were grown in modified Rogosa medium supplemented with a given carbohydrate (final concentration 0.5%) and optical densities (OD at 600 nm) were recorded at regular intervals during 24 hours. In order to evaluate the phenotypic patterns of such strains, a lower limit OD of 0.3 was used as a cut-off value to discriminate between carbohydrates that did or did not support growth of a given strain. A further *in silico* gene-trait matching excercise was performed in order to correlate an observed carbohydrate-linked growth phenotype with the presence/absence of particular genes. For this analysis all shared gene families as obtained from the comparative genomic analysis described above were organized in 51 clusters, according to their presence in each strain. Subsequently all the data (phenotypic and genomic) were binarized and compared on an individual basis. The resulting matching distances were then reported in a heatmap and manually inspected with the additional support of PFAM database (http://pfam.sanger.ac.uk).

### Construction of ***B. breve***JCM7017 insertion mutant

In order to verify our predictions from the gene trait matching an insertion mutant was created in the alcohol dehydrogenase encoding gene of the predicted sorbitol utilization gene cluster of *B. breve* JCM7017. An internal fragment of open reading frame B7017_1848 (corresponding to codon numbers 78 through to 175 out of the 335 codons present in B7017_1848) were amplified by PCR using *B. breve* JCM 7017 chromosomal DNA as a template and primer pairs IM1848F (5’-CCTAC*AAGCTT*CAGAAGTCACCAACGTCAAG-3’) and IM1848R (5’-CGATGC*TCTAGA*GATTCCGGCAAGATCCACCTG-3’) The insertion mutation was generated as described previously [24] to produce *B. breve* JCM7017-1848. Site-specific recombination in potential Tet-resistant mutant isolates was confirmed by colony PCR using primer combinations tetWFw (5’-ATGCTCATGTACGGTAAG-3’) and tetWRv (5’-CATTACCTTCTGAAACATA-3’) to verify *tetW* gene integration, and primers 1848-F (5’-GCTCCGCTGCCGCAGTTCCG-3’, positioned upstream of the selected internal fragment of B7017_1848), in combination with tetWFw to confirm integration at the correct chromosomal location.

### Nucleotide sequence accession numbers

All the sequences here generated have been submitted to GenBank database with the following accession numbers: *B. breve* 689b [GenBank: CP006715], *B. breve* 12L [GenBank: CP006711], *B. breve* 2L [GenBank: AWUG00000000], *B. breve* 31L [GenBank: AWUF00000000], *B. breve* NCFB 2258 [GenBank: CP006714], *B. breve* S27 [GenBank: CP006716], *B. breve* JCM 7017 [GenBank: CP006712], *B. breve* JCM 7019 [GenBank: CP006713].

All the sequences used for our analysis have been retrieved from GenBank database with the following accession numbers: *B. breve* UCC2003 [GenBank: NC_020517], *B. bifidum* PRL2010 [GenBank: NC_014638], *B. breve* ACS-071-V-Sch8b [GenBank: NC_017218], *B. breve* CECT 7263 [GenBank: AFVV01000000], *B. breve* DPC 6330 [GenBank: AFXX00000000], *B. breve* DSM 20213 [GenBank: ACCG00000000]; *B. longum* subsp. *longum* NCC2705 [GenBank: NC_004307], *B. longum* subsp. *longum* DJO10A [GenBank: NC_010816], *B. longum* subsp. *longum* JCM 1217 [GenBank: NC_015067], *B. longum* subsp. *longum* ATCC 15697 [GenBank: NC_017219], *B. longum* subsp. *longum* 157 F [GenBank: NC_015052], *B. longum* subsp. *longum* BBMN68 [GenBank: NC_014656], *B. longum* subsp. *infantis* ATCC 15697 [GenBank: NC_017219], *B. adolescentis* ATCC 15703 [GenBank: NC_008618], *B. dentium* Bd1 [GenBank: NC_013714], *B. animalis* subsp. *animalis* ATCC 25527 [GenBank: NC_017834], *B. animalis* subsp. *lactis* DSM 10140 [GenBank: NC_012815], *B. asteroides* PRL2011 [GenBank: NC_018720], *G. vaginalis* ATCC 14019 [GenBank: NC_014644], *L. xyli* subsp. *xyli* CTCB07 [GenBank: NC_006087], *T. whipplei* TW08/27 [GenBank: NC_004551], *Lb. plantarum* WCFS1 [GenBank: NC_004567], *C. difficile* 630 [GenBank: NC_009089].

## Electronic supplementary material

Additional file 1: Figure S1: *B. breve* mobilome. Locus map showing the presence of prophage-like elements (first three images from top) and episome (bottom figure) in *B. breve.* All the genes are coloured based on a particular (predicted) function. (PDF 71 KB)

Additional file 2: Figure S2: Whole-genome alignments. a) Dotplot alignment of eight fully sequenced *B. breve* genomes (*B. breve* UCC2003, *B. breve* 689b, *B. breve* 12L, *B. breve* NCFB 2258, *B. breve* S27, *B. breve* JCM 7017, *B. breve* JCM 7019, *B. breve* ACS-071-V-Sch8b) against the genomic sequence of *B. breve* UCC2003. (PDF 2 MB)

Additional file 3: Figure S3: *B. breve* core and dispensable genome. Cluster of orthologues classification of the gene families contained in the *B. breve* core and dispensable genome resulting from the MCL comparative analysis. As from the plot the core genome contains most of the housekeeping functions (carbohydrate and amino acid transport and metabolism, translation and biogenesis), while in the dispensable genome the higher amount of hits remains unclassified. (PDF 120 KB)

Additional file 4: Table S1: The predicted *B. breve* dispensable genome. An .xls document containing a list of gene families resulting from the comparative analysis and predicted to be included in the *B. breve* dispensable genome. (XLS 98 KB)

Additional file 5: Table S2: Gene families specific of *B. breve*. An .xls document containing a list of gene families specific of *B. breve* and absent in *B. longum*. (XLS 70 KB)

Additional file 6: Figure S5: Exopolysaccharide production clusters in *B. breve*. a) Comparative genomics of the gene clusters involved in the exopolysaccharide (EPS) production of *B. breve*. Locus map showing the distribution and similarity of EPS cluster 2 in the complete *B. breve* genomes. All the genes are coloured based on their function and percentage of similarity resulted from BLASTP alignment are also reported. b) Locus map showing the distribution and similarity of EPS cluster 1 in the complete *B. breve* genomes. All the genes are coloured based on their function and percentage of similarity resulted from BLASTP alignment are also reported. (PDF 641 KB)

Additional file 7: Figure S4: *B. breve* adhesion loci. a) Comparative genomics of the gene loci involved in the adhesion of *B. breve*. Locus map showing the distribution and similarity of the sortase-dependent pili encoding genes in *B. breve*. All the genes are coloured based on their function and percentage of similarity resulted from BLASTP alignment are also reported. b) Locus map showing the distribution and similarity of Type IV tight adherence (*tad*) locus in *B. breve*. All the genes are coloured based on their function and percentage of similarity resulted from BLASTP alignment are also reported. (PDF 510 KB)

Additional file 8: Table S3: *B. breve* supertree orthologues list. An .xls document containing a list 165 of orthologues that were used to compute the *B. breve* supertree. (XLSX 31 KB)

## Abbreviations

GIT: Gastrointestinal tract
HMOs: Human milk oligosaccharides
CGH: Comparative genome hybridazion
ORF: Open reading frame
CRISPR: Clustered regularly interspaced short palindromic repeats
R/M system: Restriction modification system
EPS: Exopolysaccharides
NGS: Next generation sequencing
IS: Insertion sequence
COG: Cluster of orthologous
GH(s): Glycosyl hydrolase(s)
FOS: Fructooligosaccharides
HCL: Hierarchical clustering.

## References

[1] Ventura M, O’Flaherty S, Claesson MJ, Turroni F, Klaenhammer TR, van Sinderen D, O’Toole PW (2009). Genome-scale analyses of health-promoting bacteria: probiogenomics. Nat Rev Microbiol.

[2] Ventura M, Turroni F, van Sinderen D (2012). Probiogenomics as a tool to obtain genetic insights into adaptation of probiotic bacteria to the human gut. Bioeng Bugs.

[3] Turroni F, Ventura M, Butto LF, Duranti S, O’Toole PW, Motherway MO, van Sinderen D (2013). Molecular dialogue between the human gut microbiota and the host: a *Lactobacillus* and *Bifidobacterium* perspective. Cell Mol Life Sci.

[4] Ventura M, Canchaya C, Tauch A, Chandra G, Fitzgerald GF, Chater KF, van Sinderen D (2007). Genomics of *Actinobacteria*: tracing the evolutionary history of an ancient phylum. Microbiol Mol Biol Rev.

[5] Turroni F, van Sinderen D, Ventura M (2011). Genomics and ecological overview of the genus *Bifidobacterium*. Int J Food Microbiol.

[6] Turroni F, Peano C, Pass DA, Foroni E, Severgnini M, Claesson MJ, Kerr C, Hourihane J, Murray D, Fuligni F, Gueimonde M, Margolles A, De Bellis G, O’Toole PW, Van Sinderen D, Marchesi JR, Ventura M (2012). Diversity of Bifidobacteria within the Infant Gut Microbiota. PLoS One.

[7] Sela DA, Chapman J, Adeuya A, Kim JH, Chen F, Whitehead TR, Lapidus A, Rokhsar DS, Lebrilla CB, German JB, Prince NP, Richardson PM, Mills DA (2008). The genome sequence of *Bifidobacterium longum* subsp. *infantis* reveals adaptations for milk utilization within the infant microbiome. Proc Natl Acad Sci USA.

[8] O’Connell Motherway M, Zomer A, Leahy SC, Reunanen J, Bottacini F, Claesson MJ, O’Brien F, Flynn K, Casey PG, Munoz JA, Kearney B, Houston AM, O’Mahony C, Higgins DG, Shanahan F, Palva A, de Vos WM, Fitzgerald GF, Ventura M, O’Toole PW, van Sinderen D (2011). Functional genome analysis *of Bifidobacterium breve* UCC2003 reveals type IVb tight adherence (Tad) pili as an essential and conserved host-colonization factor. Proc Natl Acad Sci USA.

[9] Turroni F, Serafini F, Foroni E, Duranti S, O’Connell Motherway M, Taverniti V, Mangifesta M, Milani C, Viappiani A, Roversi T, Sánchez B, Santoni A, Gioiosa L, Ferrarini A, Delledonne M, Margolles A, Piazza L, Palanza P, Bolchi A, Guglielmetti S, van Sinderen D, Ventura M (2013). Role of sortase-dependent pili of *Bifidobacterium bifidum* PRL2010 in modulating bacterium-host interactions. Proc Natl Acad Sci USA.

[10] Tettelin H, Masignani V, Cieslewicz MJ, Donati C, Medini D, Ward NL, Angiuoli SV, Crabtree J, Jones AL, Durkin AS, Deboy RT, Davidsen TM, Mora M, Scarselli M, Margarit y Ros I, Peterson JD, Hauser CR, Sundaram JP, Nelson WC, Madupu R, Brinkac LM, Dodson RJ, Rosovitz MJ, Sullivan SA, Daugherty SC, Haft DH, Selengut J, Gwinn ML, Zhou L, Zafar N (2005). Genome analysis of multiple pathogenic isolates of *Streptococcus agalactiae*: implications for the microbial “pan-genome”. Proc Natl Acad Sci USA.

[11] Medini D, Donati C, Tettelin H, Masignani V, Rappuoli R (2005). The microbial pan-genome. Curr Opin Genet Dev.

[12] Bottacini F, Medini D, Pavesi A, Turroni F, Foroni E, Riley D, Giubellini V, Tettelin H, van Sinderen D, Ventura M (2010). Comparative genomics of the genus *Bifidobacterium*. Microbiology.

[13] Ventura M, Turroni F, Zomer A, Foroni E, Giubellini V, Bottacini F, Canchaya C, Claesson MJ, He F, Mantzourani M, Mulas L, Ferrarini A, Gao B, Delledonne M, Henrissat B, Coutinho P, Oggioni M, Gupta RS, Zhang Z, Beighton D, Fitzgerald GF, O’Toole PW, van Sinderen D (2009). The *Bifidobacterium dentium* Bd1 genome sequence reflects its genetic adaptation to the human oral cavity. PLoS Genet.

[14] Turroni F, Bottacini F, Foroni E, Mulder I, Kim JH, Zomer A, Sanchez B, Bidossi A, Ferrarini A, Giubellini V, Delledonne M, Henrissat B, Coutinho P, Oggioni M, Fitzgerald GF, Mills D, Margolles A, Kelly D, van Sinderen D, Ventura M (2010). Genome analysis of *Bifidobacterium bifidum* PRL2010 reveals metabolic pathways for host-derived glycan foraging. Proc Natl Acad Sci USA.

[15] Bottacini F, Turroni F, Viappiani A, Milani C, Serafini F, Foroni E, van Sinderen D, Ventura M (2012). The genome sequences of *Bifidobacterium asteroides* PRL2011 reveals respiratory metabolic capabilities. PloS One.

[16] Makarova K, Slesarev A, Wolf Y, Sorokin A, Mirkin B, Koonin E, Pavlov A, Pavlova N, Karamychev V, Polouchine N, Shakhova V, Grigoriev I, Lou Y, Rohksar D, Lucas S, Huang K, Goodstein DM, Hawkins T, Plengvidhya V, Welker D, Hughes J, Goh Y, Benson A, Baldwin K, Lee JH, Díaz-Muñiz I, Dosti B, Smeianov V, Wechter W, Barabote R (2006). Comparative genomics of the lactic acid bacteria. Proc Natl Acad Sci USA.

[17] Siguier P, Varani A, Perochon J, Chandler M (2012). Exploring bacterial insertion sequences with ISfinder: objectives, uses, and future developments. Methods Mol Biol.

[18] Ventura M, Lee JH, Canchaya C, Zink R, Leahy S, Moreno-Munoz JA, O’Connell-Motherway M, Higgins D, Fitzgerald GF, O’Sullivan DJ, van Sinderen D (2005). Prophage-like elements in bifidobacteria: insights from genomics, transcription, integration, distribution, and phylogenetic analysis. Appl Environ Microbiol.

[19] O’Riordan K, Fitzgerald GF (1999). Molecular characterisation of a 5.75-kb cryptic plasmid from *Bifidobacterium breve* NCFB 2258 and determination of mode of replication. FEMS Microbiol Lett.

[20] Lee JH, O’Sullivan DJ (2010). Genomic insights into bifidobacteria. Microbiol Mol Biol Rev.

[21] Brouwer MS, Roberts AP, Mullany P, Allan E (2012). In silico analysis of sequenced strains of *Clostridium difficile* reveals a related set of conjugative transposons carrying a variety of accessory genes. Mob Genet Elements.

[22] Ventura M, Canchaya C, Del Casale A, Dellaglio F, Neviani E, Fitzgerald GF, van Sinderen D (2006). Analysis of bifidobacterial evolution using a multilocus approach. Int J Syst Evol Microbiol.

[23] Fanning S, Hall LJ, Cronin M, Zomer A, MacSharry J, Goulding D, Motherway MO, Shanahan F, Nally K, Dougan G, van Sinderen D (2012). Bifidobacterial surface-exopolysaccharide facilitates commensal-host interaction through immune modulation and pathogen protection. Proc Natl Acad Sci USA.

[24] O’Connell Motherway M, O’Driscoll J, Fitzgerald GF, Van Sinderen D (2009). Overcoming the restriction barrier to plasmid transformation and targeted mutagenesis in *Bifidobacterium breve* UCC2003. Microb Biotechnol.

[25] Foroni E, Serafini F, Amidani D, Turroni F, He F, Bottacini F, O’Connell Motherway M, Viappiani A, Zhang Z, Rivetti C, van Sinderen D, Ventura M (2011). Genetic analysis and morphological identification of pilus-like structures in members of the genus *Bifidobacterium*. Microb Cell Fact.

[26] Zhao Y, Wu J, Yang J, Sun S, Xiao J, Yu J (2012). PGAP: pan-genomes analysis pipeline. Bioinformatics.

[27] Pokusaeva K, Neves AR, Zomer A, O’Connell-Motherway M, Macsharry J, Curley P, Fitzgerald GF, Van Sinderen D (2010). Ribose utilization by the human commensal *Bifidobacterium breve* UCC2003. Microb Biotechnol.

[28] Pokusaeva K, O’Connell-Motherway M, Zomer A, Fitzgerald GF, van Sinderen D (2009). Characterization of two novel alpha-glucosidases from *Bifidobacterium breve* UCC2003. Appl Environ Microbiol.

[29] Aslanidis C, Schmid K, Schmitt R (1989). Nucleotide sequences and operon structure of plasmid-borne genes mediating uptake and utilization of raffinose in *Escherichia coli*. J Bacteriol.

[30] Ryan SM, Fitzgerald GF, van Sinderen D (2006). Screening for and identification of starch-, amylopectin-, and pullulan-degrading activities in bifidobacterial strains. Appl Environ Microbiol.

[31] Motherway MO, Fitzgerald GF, van Sinderen D (2011). Metabolism of a plant derived galactose-containing polysaccharide by *Bifidobacterium breve* UCC2003. Microb Biotechnol.

[32] Pokusaeva K, O’Connell-Motherway M, Zomer A, MacSharry J, Fitzgerald GF, van Sinderen D (2011). Cellodextrin Utilization by *Bifidobacterium breve* UCC2003. Appl Environ Microb.

[33] Nishimoto M, Kitaoka M (2007). Identification of N-acetylhexosamine 1-kinase in the complete lacto-N-biose I/galacto-N-biose metabolic pathway in *Bifidobacterium longum*. Appl Environ Microbiol.

[34] Garrido D, Dallas DC, Mills DA (2013). Consumption of human milk glycoconjugates by infant-associated bifidobacteria: mechanisms and implications. Microbiology.

[35] Locascio RG, Ninonuevo MR, Kronewitter SR, Freeman SL, German JB, Lebrilla CB, Mills DA (2009). A versatile and scalable strategy for glycoprofiling bifidobacterial consumption of human milk oligosaccharides. Microb Biotechnol.

[36] Ward RE, Ninonuevo M, Mills DA, Lebrilla CB, German JB (2007). In vitro fermentability of human milk oligosaccharides by several strains of bifidobacteria. Mol Nutr Food Res.

[37] Kim JH, An HJ, Garrido D, German JB, Lebrilla CB, Mills DA (2013). Proteomic analysis of Bifidobacterium longum subsp. infantis reveals the metabolic insight on consumption of prebiotics and host glycans. PloS one.

[38] Falony G, Vlachou A, Verbrugghe K, De Vuyst L (2006). Cross-feeding between *Bifidobacterium longum* BB536 and acetate-converting, butyrate-producing colon bacteria during growth on oligofructose. Appl Environ Microbiol.

[39] Coutinho PM, Henrissat B (1999). Life with no sugars?. J Mol Microbiol Biotechnol.

[40] Hirayama Y, Sakanaka M, Fukuma H, Murayama H, Kano Y, Fukiya S, Yokota A (2012). Development of a double-crossover markerless gene deletion system in *Bifidobacterium longum*: functional analysis of the alpha-galactosidase gene for raffinose assimilation. Appl Environ Microbiol.

[41] O’Connell KJ, O’Connell Motherway M, O’Callaghan J, Fitzgerald GF, Ross RP, Ventura M, Stanton C, van Sinderen D (2013). Metabolism of four alpha-glycosidic linkage-containing oligosaccharides by *Bifidobacterium breve* UCC2003. Appl Environ Microbiol.

[42] Turroni F, Foroni E, Giubellini V, Ribbera A, Merusi P, Cagnasso P, Bizzarri B, De’ Angelis GL, Shanahan F, van Sinderen D: **Exploring the diversity of the bifidobacterial population in the human intestinal tract.** *Appl Environ Microbiol* **75:**1534–1545. 10.1128/AEM.02216-08 PMC2655441 19168652

[43] Milani C, Duranti S, Lugli GA, Bottacini F, Strati F, Arioli S, Foroni E, Turroni F, van Sinderen D, Ventura M (2013). Comparative genomics of Bifidobacterium animalis subsp. lactis reveals a strict monophyletic bifidobacterial taxon. Appl Environ Microbiol.

[44] Patel RK, Jain M (2012). NGS QC Toolkit: a toolkit for quality control of next generation sequencing data. PloS one.

[45] Altschul SF, Gish W, Miller W, Myers EW, Lipman DJ (1990). Basic local alignment search tool. J Mol Biol.

[46] Tatusov RL, Galperin MY, Natale DA, Koonin EV (2000). The COG database: a tool for genome-scale analysis of protein functions and evolution. Nucleic Acids Res.

[47] Schattner P, Brooks AN, Lowe TM (2005). The tRNAscan-SE, snoscan and snoGPS web servers for the detection of tRNAs and snoRNAs. Nucleic Acids Res.

[48] Lagesen K, Hallin P, Rodland EA, Staerfeldt HH, Rognes T, Ussery DW (2007). RNAmmer: consistent and rapid annotation of ribosomal RNA genes. Nucleic Acids Res.

[49] Rice P, Longden I, Bleasby A (2000). EMBOSS: the European Molecular Biology Open Software Suite. Trends Genet.

[50] Kurtz S, Phillippy A, Delcher AL, Smoot M, Shumway M, Antonescu C, Salzberg SL (2004). Versatile and open software for comparing large genomes. Genome Biol.

[51] Enright AJ, Van Dongen S, Ouzounis CA (2002). An efficient algorithm for large-scale detection of protein families. Nucleic Acids Res.

[52] Thompson JD, Gibson TJ, Higgins DG (2002). Multiple sequence alignment using ClustalW and ClustalX. Current protocols in bioinformatics/editoral board, Andreas D Baxevanis [et al.].

[53] Guindon S, Gascuel O (2003). A simple, fast, and accurate algorithm to estimate large phylogenies by maximum likelihood. Syst Biol.

