# Supplementary figures and images for: Comparative genomics of the Bifidobacterium breve taxon
Source: BMC Genomics. 2014 Mar 1;15(1):170. doi: 10.1186/1471-2164-15-170 (PMC4007704; doi:10.1186/1471-2164-15-170)

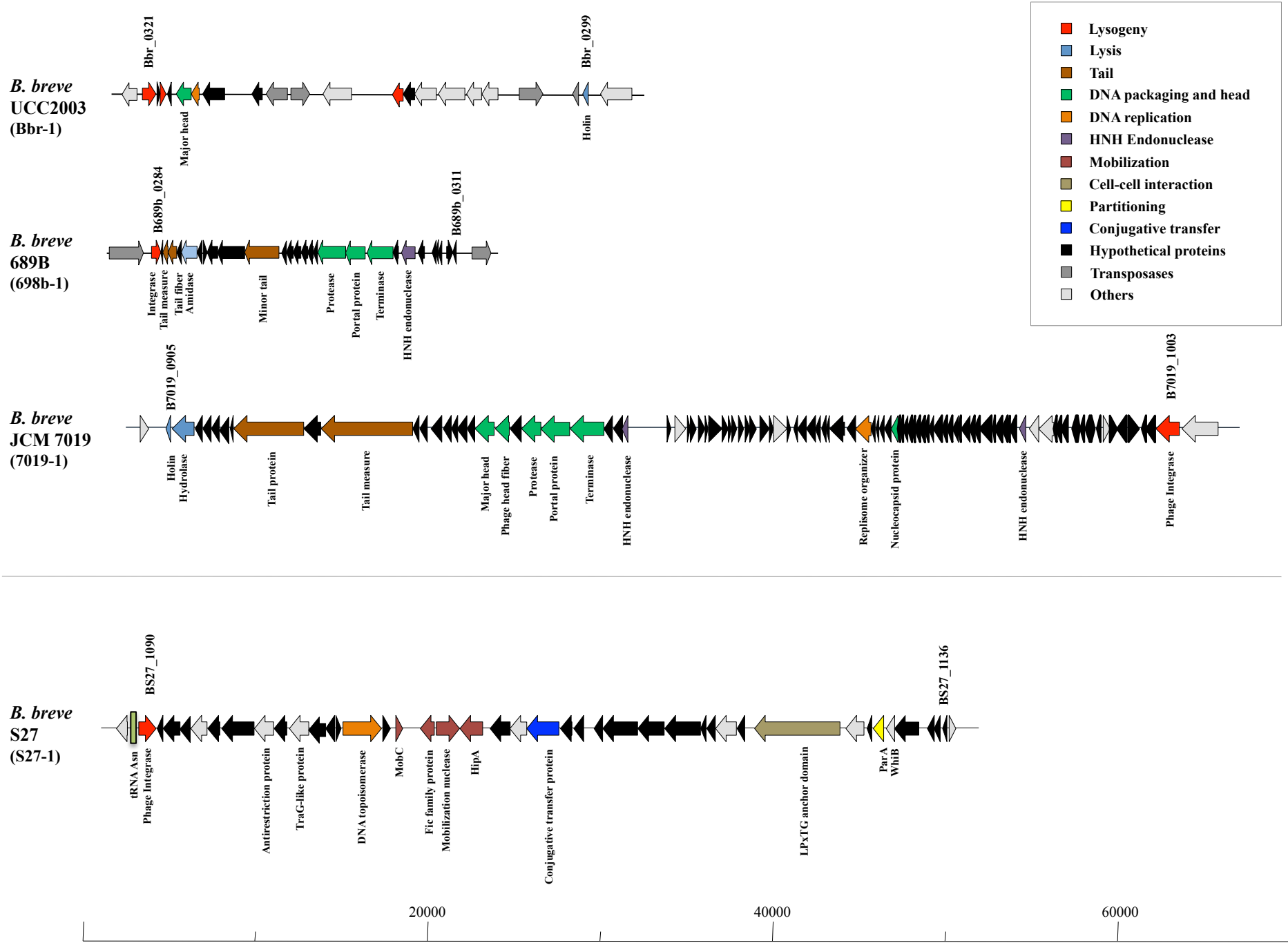

Supplement: Supplementary file 1 — Additional file 1: Figure S1: B. breve mobilome. Locus map showing the presence of prophage-like elements (first three images from top) and episome (bottom figure) in B. breve. All the genes are coloured based on a particular (predicted) function. (PDF 71 KB) [file 12864_2013_7017_MOESM1_ESM.pdf]

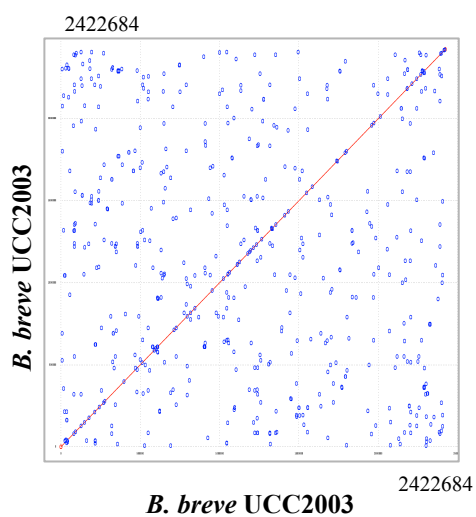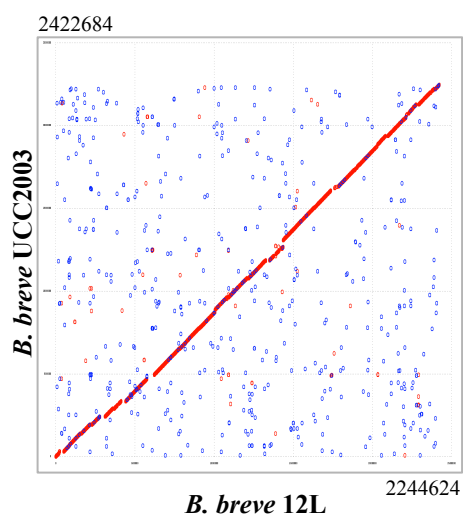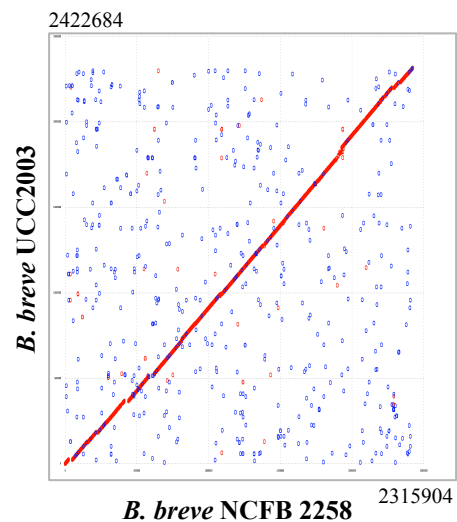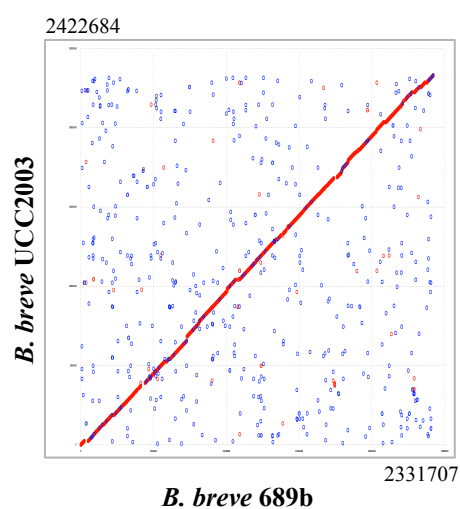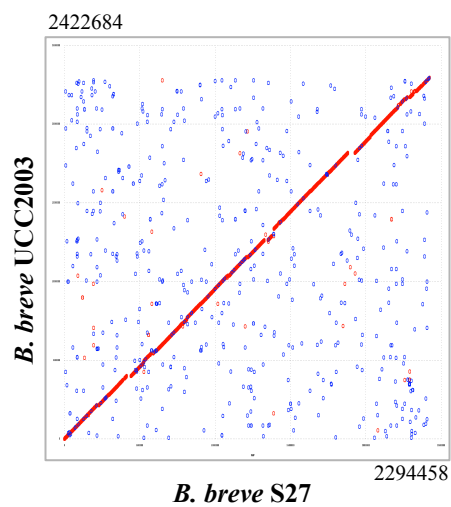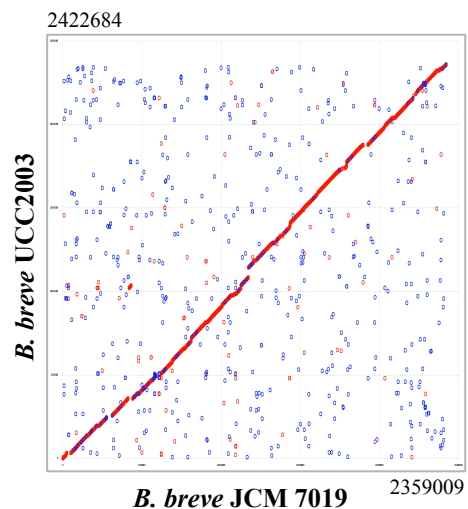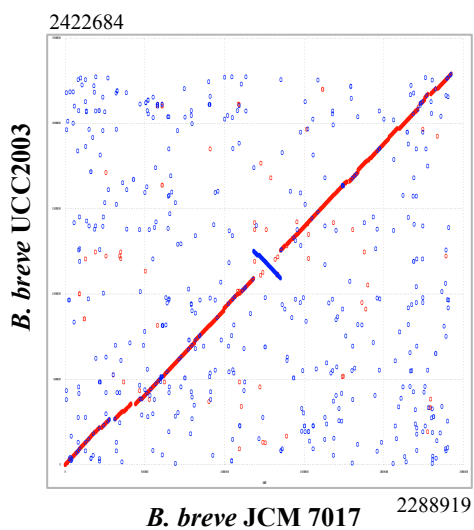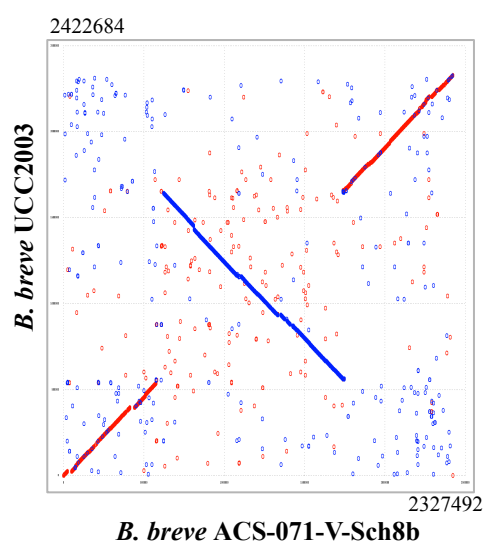

Supplement: Supplementary file 2 — Additional file 2: Figure S2: Whole-genome alignments. a) Dotplot alignment of eight fully sequenced B. breve genomes (B. breve UCC2003, B. breve 689b, B. breve 12L, B. breve NCFB 2258, B. breve S27, B. breve JCM 7017, B. breve JCM 7019, B. breve ACS-071-V-Sch8b) against the genomic sequence of B. breve UCC2003. (PDF 2 MB) [file 12864_2013_7017_MOESM2_ESM.pdf]

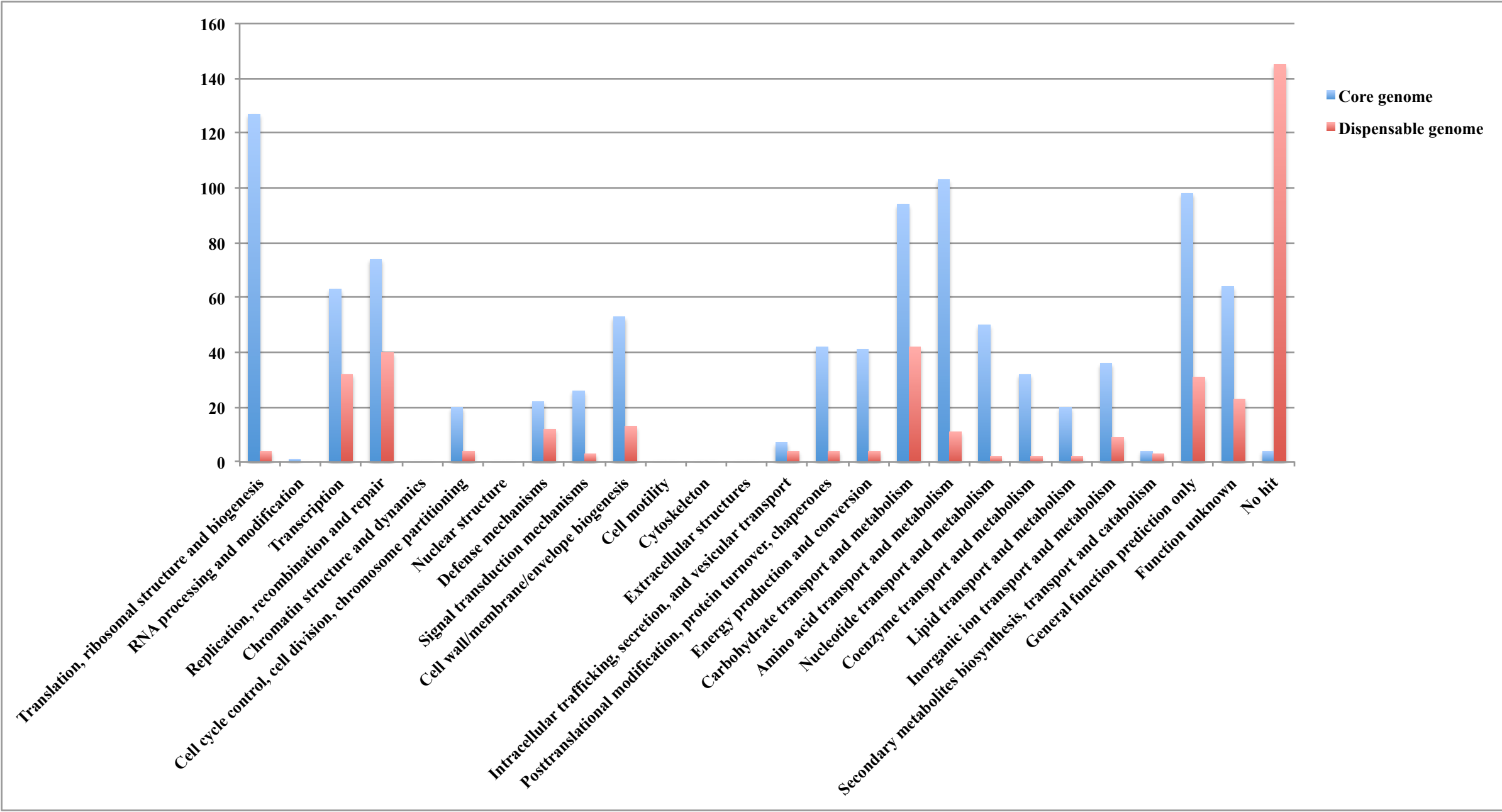

Supplement: Supplementary file 3 — Additional file 3: Figure S3: B. breve core and dispensable genome. Cluster of orthologues classification of the gene families contained in the B. breve core and dispensable genome resulting from the MCL comparative analysis. As from the plot the core genome contains most of the housekeeping functions (carbohydrate and amino acid transport and metabolism, translation and biogenesis), while in the dispensable genome the higher amount of hits remains unclassified. (PDF 120 KB) [file 12864_2013_7017_MOESM3_ESM.pdf]

## EPS Cluster 2

a)

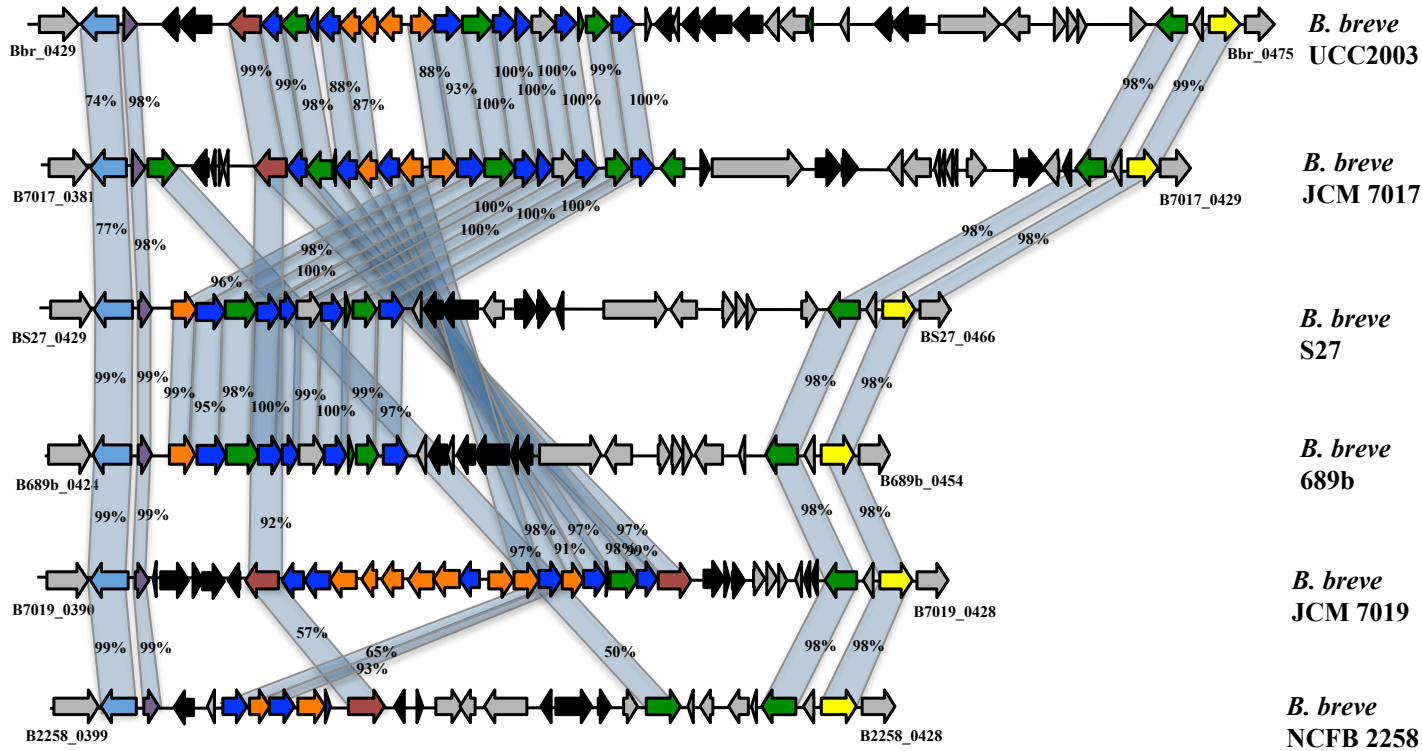

## EPS Cluster 1

b)

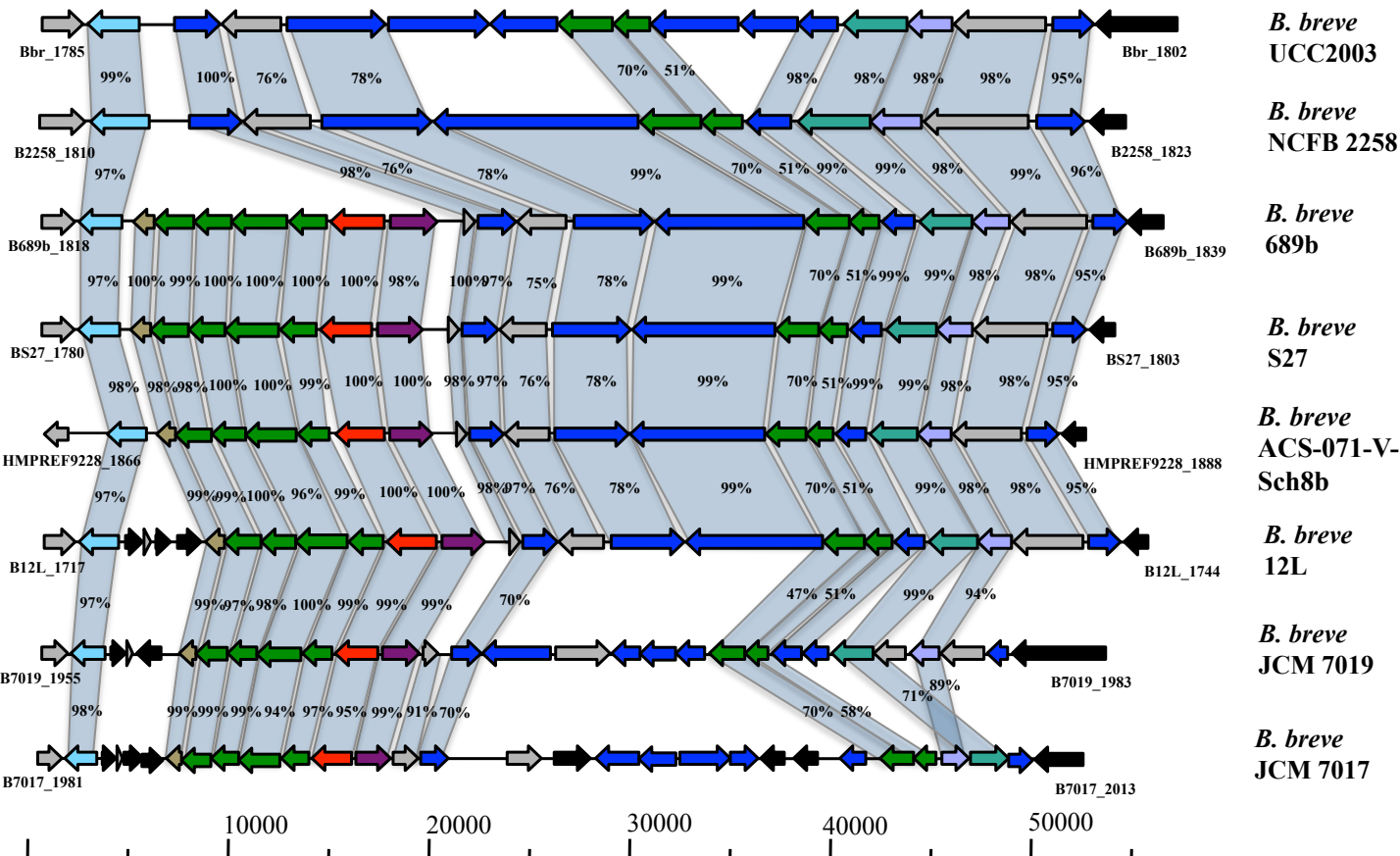

Supplement: Supplementary file 6 — Additional file 6: Figure S5: Exopolysaccharide production clusters in B. breve. a) Comparative genomics of the gene clusters involved in the exopolysaccharide (EPS) production of B. breve. Locus map showing the distribution and similarity of EPS cluster 2 in the complete B. breve genomes. All the genes are coloured based on their function and percentage of similarity resulted from BLASTP alignment are also reported. b) Locus map showing the distribution and similarity of EPS cluster 1 in the complete B. breve genomes. All the genes are coloured based on their function and percentage of similarity resulted from BLASTP alignment are also reported. (PDF 641 KB) [file 12864_2013_7017_MOESM6_ESM.pdf]

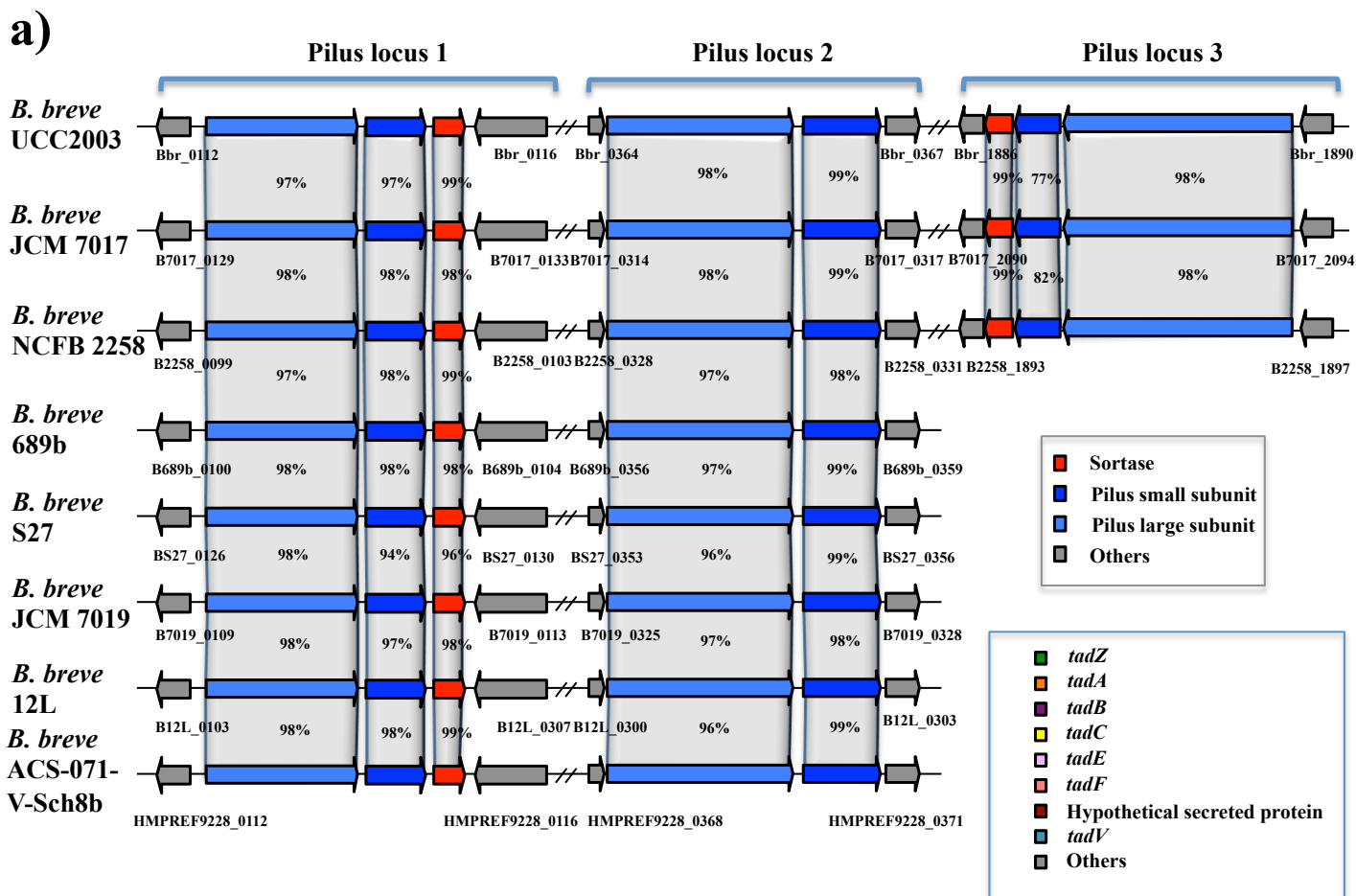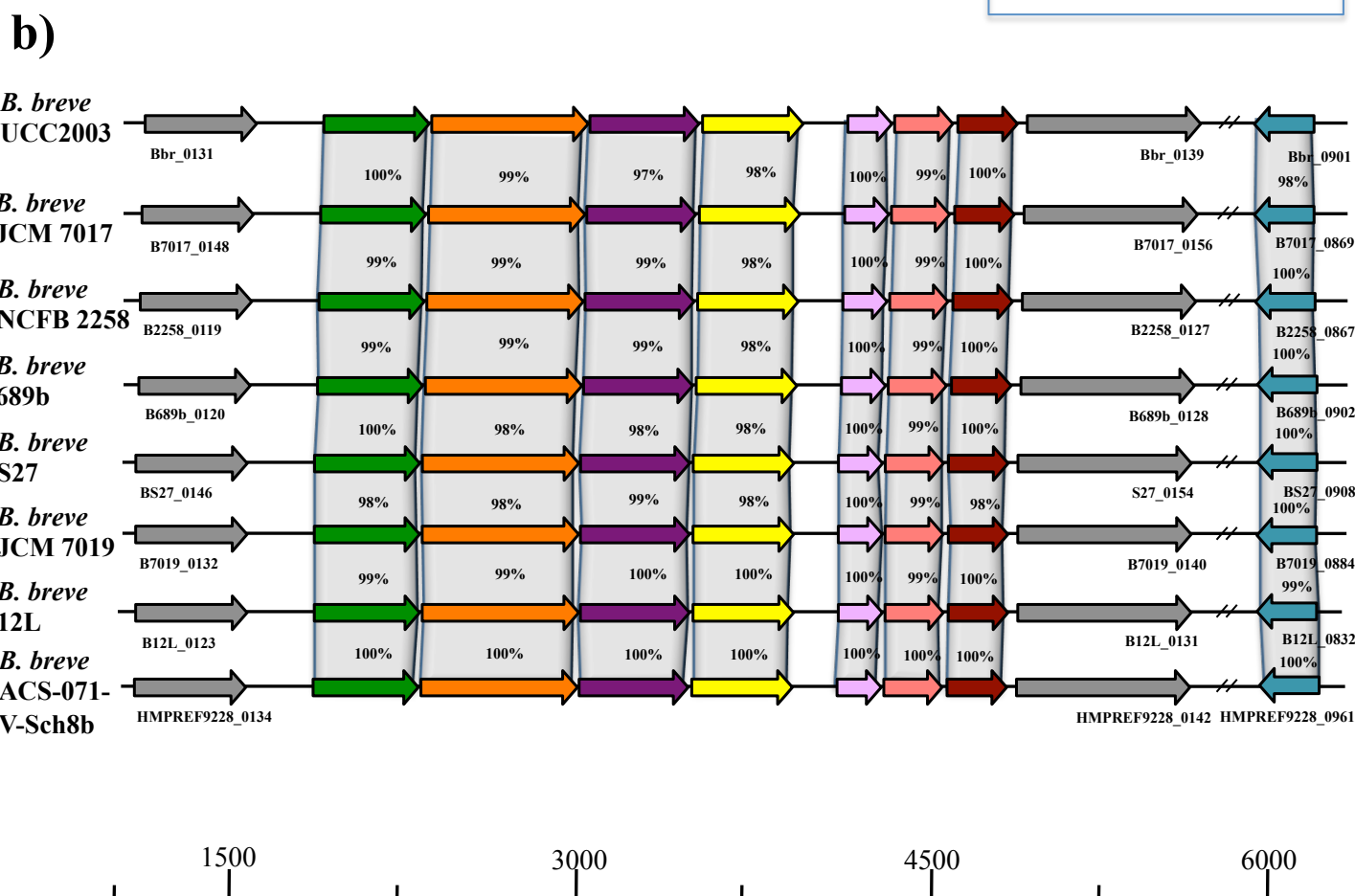

Supplement: Supplementary file 7 — Additional file 7: Figure S4: B. breve adhesion loci. a) Comparative genomics of the gene loci involved in the adhesion of B. breve. Locus map showing the distribution and similarity of the sortase-dependent pili encoding genes in B. breve. All the genes are coloured based on their function and percentage of similarity resulted from BLASTP alignment are also reported. b) Locus map showing the distribution and similarity of Type IV tight adherence (tad) locus in B. breve. All the genes are coloured based on their function and percentage of similarity resulted from BLASTP alignment are also reported. (PDF 510 KB) [file 12864_2013_7017_MOESM7_ESM.pdf]
